# Succession of Gut Microbial Structure in Twin Giant Pandas During the Dietary Change Stage and Its Role in Polysaccharide Metabolism

**DOI:** 10.3389/fmicb.2020.551038

**Published:** 2020-09-22

**Authors:** Mingye Zhan, Lei Wang, Chunyu Xie, Xiaohua Fu, Shu Zhang, Aishan Wang, Yingmin Zhou, Chunzhong Xu, Hemin Zhang

**Affiliations:** ^1^College of Environmental Science and Engineering, Institute of Pollution Control and Ecological Safety, Tongji University, Shanghai, China; ^2^Shanghai Wild Animal Park Development Co., Ltd., Shanghai, China; ^3^Shanghai Zoo, Shanghai, China; ^4^China Conservation and Research Centre for the Giant Panda, Dujiangyan, China

**Keywords:** gut microbes, succession, *Clostridium*, giant panda cub, cellulose degradation, functional gene prediction, GH5

## Abstract

Adaptation to a bamboo diet is an essential process for giant panda growth, and gut microbes play an important role in the digestion of the polysaccharides in bamboo. The dietary transition in giant panda cubs is particularly complex, but it is an ideal period in which to study the effects of gut microbes on polysaccharide use because their main food changes from milk to bamboo (together with some bamboo shoot and coarse pastry). Here, we used 16S rDNA and internal transcribed spacer 1 (ITS1) DNA sequencing and metagenomic sequencing analysis to investigate the succession of the gut microbial structure in feces sampled from twin giant panda cubs during the completely dietary transition and determine the abundances of polysaccharide-metabolizing genes and their corresponding microbes to better understand the degradation of bamboo polysaccharides. Successive changes in the gut microbial diversity and structure were apparent in the growth of pandas during dietary shift process. Microbial diversity increased after the introduction of supplementary foods and then varied in a complex way for 1.5–2 years as bamboo and complex food components were introduced. They then stabilized after 2 years, when the cubs consumed a specialized bamboo diet. The microbes had more potential to metabolize the cellulose in bamboo than the hemicellulose, providing genes encoding cellulase systems corresponding to glycoside hydrolases (GHs; such as GH1, GH3, GH5, GH8, GH9, GH74, and GH94). The cellulose-metabolizing species (or genes) of gut bacteria was more abundant than that of gut fungi. Although cellulose-metabolizing species did not predominate in the gut bacterial community, microbial interactions allowed the giant pandas to achieve the necessary dietary shift and ultimately adapt to a bamboo diet.

## Introduction

The giant panda, *Ailuropoda melanoleuca*, is the flagship species of biodiversity conservation throughout the world (Wei et al., 2019). It belongs to the order Carnivora and has a typical carnivorous intestinal structure (Zhang et al., 2007). However, it consumes a high-cellulose diet composed of bamboo (> 90%) and complementary foods (Hu et al., 2017).

Researchers have found no cellulolytic enzyme genes in the genome of the giant panda, which must rely instead on its gut microbes to digest cellulose (Zhang et al., 2007; Li et al., 2010; Wei et al., 2019). However, it lacks a herbivore-like rumen, in which more than half the carbohydrate-active proteins can degrade cellulose and many still-unknown bacterial species (and candidate genes) participate in the relevant activities (Hess et al., 2011; Neumann and Suen, 2018; Sheikh et al., 2019). Several microbial strains and GH genes that can degrade cellulose have been identified with high-throughput sequencing and culture *in vitro* (Anand et al., 2012; Mearls and Lynd, 2014; Wang et al., 2016; Guo W. et al., 2018; Liu et al., 2019). These findings demonstrate, to some extent, the important status of cellulose in the nutrient usage of the giant panda. The giant panda has evolved a pseudothumb for grasping bamboo and metabolic pathways to efficiently utilize the essential amino acids, fatty acids, and vitamins in bamboo (Hu et al., 2017; Guo M. et al., 2018) and has also established close interactions with specific gut microbes during its adaptation to a bamboo diet (Mckenney et al., 2017), not only for nutrient digestion but also for detoxification (Zhu et al., 2018). Zhang et al. (2018) suggested that the intestinal microbes of the giant panda digest cellulose poorly and that the hemicellulose in their food is mainly utilized by giant panda cubs. However, hemicellulose is usually more stable than cellulose and harder to degrade (Bhat et al., 2011; Zhang et al., 2018).

Most of these studies have been based on the analysis of gut microbial diversity and microbial genomes. These maybe because the digestive capacity of the gut microbes in the giant panda for bamboo cellulose and hemicellulose is difficult to analyze directly with enzyme activity tests. Both cellulases and hemicellulases are multi-enzyme systems (Zhang et al., 2019). Microorganisms can degrade cellulose while degrading small amounts of hemicellulose and that the biodegradation of hemicellulose by microbes is usually accompanied by cellulose degradation (Du et al., 2019; Luo et al., 2019). Changes in the structure of polysaccharide-metabolizing microbes and their functional genes with high-throughput sequencing may more clearly and systematically reflect microbial potential for degradation of different polysaccharides in panda (Hess et al., 2011; Berlemont et al., 2020), although these studies have so far failed to present a unified statement with different sampling techniques and analytical methods. For instance, some of the studies mentioned above were comparative studies of the cellulose-digesting ability of herbivores and adult giant pandas. However, the intestinal physiological structures and feeding behaviors of these animals vary greatly (Guo W. et al., 2018). Other studies have identified the changes in the gut flora that occur in different individual pandas from cubs to the juvenile and their functional genes much greater potential for encoding hemicellulases than cellulases (Zhang et al., 2018). However, the microbial contributors to polysaccharide-metabolizing genes are still unclear and the bias introduced by the use of different individuals with different physiological and dietary conditions has not been accommodated.

Giant panda cubs undergo a specific process of dietary change when they transition from milk to a high-polysaccharide bamboo diet in their adaptation to a specialized bamboo diet. This process is especially important to panda cubs, and during it, they are very fragile, with a low survival rate (Guo M. et al., 2018; Stewart et al., 2018). It is quite a complex period, but it is ideal for investigating the relationships between intestinal microbes and the polysaccharide metabolism of the giant panda. Zhang et al. (2018) have demonstrated that the gut microbes of suckling panda cubs stabilized after the age of 1 year and that the bacterial population structure in suckling cubs differed from that in adult pandas on a bamboo diet. However, their analysis of different individuals is likely to have amplified the impact of individual differences and discontinuous sampling, which will have confused the complete succession of gut microbes that occurs during adaptation to bamboo. Giant panda cubs still consume a little milk after the age of 1 year, so whether a stable microbial structure is established in that period is still unclear. Whether cellulose or hemicellulose hopefully becomes the preferential substrate of gut microbes for panda still needs investigation.

Here, we assume that (i) the gut microbial structure of giant panda cubs still changes considerably after they are 1 year old and gradually adopts the stable microbial components of the adult giant panda, which should correspond to dietary shift and bamboo adaptation; (ii) the types and abundances of polysaccharide-metabolizing genes will change with the succession of complex gut microbial community, forming microbial interaction and potentially functional gene groups encoding cellulases and hemicellulases to deal with the intractable polysaccharides in bamboo. To test these hypotheses, we investigated the gut microbial structures in twin giant panda cubs reared with similar physiological and growth characteristics, to circumvent any individual or environmental differences, from when they mainly consumed formula milk until their adaptation to a fully bamboo diet. In this way, we studied (i) the succession of the gut microbial diversity and components during the period of dietary change, (ii) the types and abundance of functional genes encoding polysaccharides metabolism, and (iii) the identities of the microbes and their potential strategies that contribute to polysaccharide metabolism in giant panda.

## Materials and Methods

### Giant Panda Breeding and Sample Collection

To investigate the succession of intestinal microbes in giant panda cubs during dietary change and their adaptation to a bamboo diet, two captive-born giant pandas bred in the Shanghai Wild Animal Park and born in October 2016 were included in the study (Supplementary Table S1). The twin panda cubs were named YueYue and BanBan. Sampling began in August 2017 and continued to February 2019 (once every 1–3 months, a total of 11 times, details in Supplementary Material). Because their diet varied as they grew, the pandas’ feces were divided into four stages (Table 1). The health status of both pandas was monitored during the sampling period and no abnormalities were detected. Fresh fecal samples were collected immediately each morning and frozen (−20°C) in the laboratory before DNA extraction.

**TABLE 1:** Basic feeding information of panda cubs during dietary change.

Period number	Developmental stage	Food components	Stool type	Stool weight
S1	10 months to 1 year old	Milk as the main food (breast milk and artificial milk 0.76–0.86 kg), supplemented with a bit of bamboo shoots (0.4–0.5 kg)	No bamboo found in the feces	0–0.2 kg/day
S2	1–1.5 years old	Milk decreased and bamboo increased (artificial milk 1.0–0.45 kg; developed the ability to eat bamboo skillfully), supplemented with a bit of bamboo shoots (0.8–1.2 kg) and coarse pastry (0.05–0.1 kg), carrot (0.05–0.24 kg), and apple (0.02–0.07 kg)	Bamboo feces forming gradually	0.2–10 kg/day
S3	1.5–2 years old	Bamboo as the main food (artificial milk down to 0 kg after Aug 2018; bamboo 10.8–19.5 kg), supplemented with a bit of bamboo shoots (from 1.2 up to 4.0 kg until Aug 2018, then down to 2.0 kg) and coarse pastry (0.2–0.6 kg), carrot (0.24–0.3 kg), and apple (0.1–0.3 kg)	Bamboo feces	9.0–16.2 kg/day
S4	After 2 years old	Bamboo as the main food (bamboo 16.9–25.6 kg), supplemented with a bit of bamboo shoots (2.5–3.0 kg) and coarse pastry (0.7–0.9 kg), carrot (0.3 kg), and apple (0.3 kg)	Bamboo feces	14–21.2 kg/day

### DNA Extraction, Sequencing, and 16S rDNA Analysis

The frozen stool samples were thawed on ice packs. Equal contamination-free core parts of each sample (10 g/day) were taken from five stool samples collected on five consecutive days with a clean, sterile pharmacy spoon and mixed for later analysis. The DNA was extracted from the giant panda fecal samples with the PowerSoil^®^ DNA Isolation Kit (Mo Bio Laboratories Inc., Carlsbad, CA, United States), according to the manufacturer’s instructions. The extracted DNA was stored at −20°C before analysis. A pyrosequencing analysis was performed on the Illumina Miseq PE300 platform by Shanghai Majorbio Technology Co., Ltd. (Shanghai, China). The details of the DNA extraction steps, 16 S, and ITS-1 sequence amplification are available in Supplementary Material. The raw reads were deposited into the NCBI Sequence Read Archive (SRA) database (Accession Number: PRJNA626529).

The raw fastq files were demultiplexed and quality-filtered by fastp version 0.19.6 (Chen et al., 2018) and merged by FLASH (version 1.2.11)^1^ (Magoč and Salzberg, 2011), according to the following criteria: (i) the 300 bp reads were truncated at any site receiving an average quality score of <20 over a 50 bp sliding window, and the truncated reads shorter than 50 bp were discarded, reads containing ambiguous characters were also discarded; (ii) only overlapping sequences longer than 10 bp were assembled according to their overlapped sequence. The maximum mismatch ratio of overlap region was 0.2. Reads that could not be assembled were discarded; (iii) samples were distinguished according to the barcode and primers, and the sequence direction was adjusted, exact barcode matching, two-nucleotide mismatch in primer matching. The operational taxonomic units (OTUs) were clustered with a 97% similarity cutoff using UPARSE (version 7.1)^2^ (Edgar et al., 2011). Chimeric sequences were identified and removed with UCHIME. The taxonomy of each OUT representative sequence was analyzed by RDP Classifier version 2.11 (Wang et al., 2007) against the 16S (Silva v132) and ITS (UNITE v8.0) database using confidence threshold of 0.7.

### Metagenomic Sequencing Analysis

Total genomic DNA was extracted from the giant panda fecal samples (samples from S1, S3, and S4, *n* = 6), and the extraction steps were the same as mentioned above in section “DNA Extraction, Sequencing, and 16S rDNA Analysis.” The concentration and purity of the extracted DNA were determined with a TBS-380 Mini-Fluorometer (Turner BioSystems, San Francisco, CA, United States) and a NanoDrop 2000 spectrophotometer (NanoDrop, Wilmington, DE, United States), respectively. DNA quality was checked on 1% agarose gel, and the DNA was then fragmented to an average size of about 400 bp using the Covaris M220 Focused ultrasonicator (Gene Company Ltd., China) to construct a paired-end library. The paired-end library was constructed using the NEXTflex^TM^ Rapid DNA-Seq Kit (Bioo Scientific, Austin, TX, United States). Adapters containing the full complement of sequencing primer hybridization sites were ligated to the blunt-ended fragments. Paired-end sequencing was performed on the Illumina NovaSeq platform (Illumina Inc., San Diego, CA, United States) at Majorbio Bio-Pharm Technology Co., Ltd. (Shanghai, China) using NovaSeq Reagent Kits, according to the manufacturer’s instructions^3^.

The data were analyzed on the free online platform Majorbio Cloud Platform^4^. The paired-end Illumina reads were trimmed of adaptors, and low-quality reads (length < 50 bp; with a quality value < 20; or containing N bases) were removed with fastp (version 0.20.0)^5^ (Chen et al., 2018). The metagenomic data were assembled with MEGAHIT (version 1.1.2)^6^ (Li et al., 2015), which makes use of succinct de Bruijn graphs. Contigs with a length of ≥300 bp were selected as the final results for assembly, and the contigs were then used for gene prediction and annotation. The open reading frames (ORFs) in each assembled contig were predicted with MetaGene^7^ (Noguchi et al., 2006). The predicted ORFs (≥100 bp) were retrieved and translated (by NCBI). A non-redundant (NR) gene catalog was constructed with CD-HIT (version 4.6.1)^8^ with 90% sequence identity and 90% coverage (Fu et al., 2012). After quality control, the remaining reads were mapped to the gene sets with SOAPaligner (version 2.21)^9^ with 95% identity (Li et al., 2008), and the gene abundance in each sample was evaluated. Representative sequences from the NR gene catalog were aligned to those in the NCBI NR database for taxonomic annotation, using Diamond (version 0.8.35)^10^ (according to best-hit method with an *e*-value cutoff of 1e-5), against the eggNOG database for cluster of orthologous groups (COG) of proteins annotation, using Diamond (version 0.8.35)^10^ (with an e-value cutoff of 1e-5), and against the Kyoto Encyclopedia of Genes and Genomes (KEGG) database for KEGG annotation, using Diamond (version 0.8.35)^10^ (with an e-value cutoff of 1e-5). The carbohydrate-active enzymes were annotated with hmmscan^11^ against the CAZy database, with an e-value cutoff of 1e-5 (Buchfink et al., 2015).

### Statistical Analysis

The data on microbial α-diversity in the panda feces were calculated with OriginPro 8 SR0 (version 8.0724). Significant differences in α-diversity between groups were detected with Welch’s *t*-test. The R language vegan package was used to draw the OTU community bar charts and heatmaps. A β-diversity distance matrix was constructed, and a hierarchical clustering analysis was performed based on it. The UPGMA algorithm was then used to build a tree structure with R. The Bray–Curtis method was used to calculate the distance between two samples and to quantify the differences in the species abundance distributions among the samples.

The OTU abundance table was standardized with Phylogenetic Investigation of Communities by Reconstruction of Unobserved States 2 (PICRUSt 2), a software package for the functional prediction of 16S amplified sequencing results, which removes the effect of the copy number of the 16S rRNA marker gene in the species genome. Each OTU corresponding to a phylogenetic lineage in this software was then used to annotate the COG and KEGG functions of the OTUs to produce the OTU annotation information for each functional level and the abundance information for each function. PICRUSt 2 was also used to analyze the fungal data.

The R language vegan package was also used to draw a heatmap of the CAZy genes [screening for GHs and cellulose-binding modules (CBMs)] based on a specific enzyme (EC 3.2.1.4, EC 3.2.1.8, and EC 3.2.1.37) gene catalog. A contribution analysis of the microbial species for polysaccharide-metabolizing enzyme genes in the metagenomic sequencing samples was based on a CAZy gene catalog (mainly GHs and CBMs), which was annotated with KEGG and identifying genes corresponding to the putative cellulases (EC3.2.1.4) and hemicellulases (EC3.2.1.8 and EC3.2.1.37), and which were annotated with NR database to identify specific functional species. The correlation analysis was based on the relative abundances of species and functional genes. Each contribution was then characterized by the size of the correlation value. Spearman’s correlation analyses were also based on the CAZy gene catalog (mainly GHs and CBMs), which contained specific genes mainly encoding putative cellulases and hemicellulases, and their corresponding species were identified by Gene ID (>25 species) in the samples (annotated with the NR database). The microbial species were accurately classified to the species level and categorized by color at the genus level. The Spearman’s correlation coefficient threshold was 0.5 (*P* < 0.05). The sample data involved in the enzyme contribution analysis described above and Spearman’s correlation analysis were used in the sample sum calculations for the same dietary shift stage, and all abundance values for species and genes were calculated as read numbers/gene length. The 50 most abundant species and genes were presented and were analyzed in this study.

More details of the experimental data are available in Supplementary Material.

## Results

### Variations in the Gut Microbial Diversity During Dietary Change and Bamboo Adaptation

**FIGURE 1:**
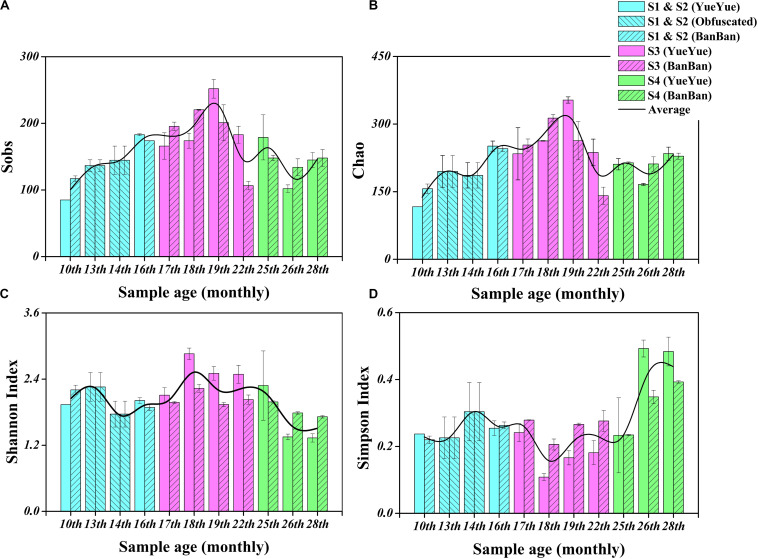
Fecal bacterial α-diversity in giant panda cubs during the dietary change stage. **(A)** S_obs_ index; **(B)** Chao index; **(C)** Shannon’s index; **(D)** Simpson’s index. Colored bars represent the different stages of dietary change. Blue bars represent individual diversity indices in stage 1 and stage 2 (formula and chewing bamboo), purple bars represent those in stage 3 (increasing bamboo consumption), and green bars represent those in stage 4 (bamboo diet). Results are expressed as means ± standard errors. Intestinal bacterial α-diversity is indicated by the height of the column at each sampling time, and the overall fluctuations in the four indices are expressed by the black trend lines.

We characterized the fecal bacterial diversity of captive twin giant panda cubs raised at the Shanghai Wild Animal Park (Shanghai, China). Both the cubs had similar physiological and growth characteristics (Supplementary Table S1). When analyzing the fecal microbial diversity in the two cubs, we observed interesting trends and successions in both α- and β-diversity (Supplementary Table S2). First, the total number of species observed (S_obs_) and the Chao diversity index, which reflect the intestinal bacterial richness, increased significantly (*P* < 0.05 on Welch’s *t-*test) with the development of both cubs from 10 months to 1.5 years, as their solid foods (such as bamboo shoots, coarse pastry, and bamboo) increased. These indices then gradually stabilized, and after the age of 2 years, when the cubs were completely weaned and predominantly ate bamboo, bacterial richness showed no significant changes (Table 1, Figures 1A,B, and Supplementary Figure S1). Shannon’s index, which indicates microbial heterogeneity, and Simpson’s index, which describes the probability that the taxa obtained in two consecutive random samples of the microbial community belong to the same species, also indicate the proportions of the dominant microbes, at least to some extent. The decline in Shannon’s index and the increase in Simpson’s index implied that the bacterial communities became less heterogeneous and contained larger proportions of the dominant taxa after the cubs were 2 years old (*P* < 0.05 on Welch’s *t-*test; Figures 1C,D and Supplementary Figure S1). Bacterial populations that were unimportant or could not adapt to gut environment might be replaced. The gut bacterial diversity of the two cubs showed significant individual differences (*P* < 0.05 on Student’s *t-*test) at 1.5–2 years old, but there were no significant individual differences at the other sampling times during the period of dietary change (Supplementary Figure S1 and Table 1).

**FIGURE 2:**
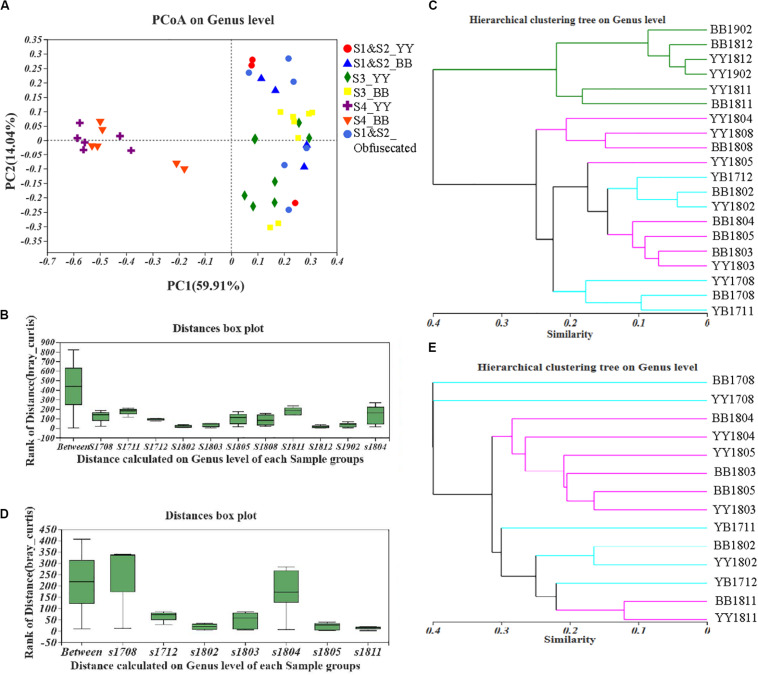
Fecal bacterial and fungal β-diversity in giant panda cubs during the period of dietary change. Colored symbols represent different stages of dietary change. **(A–C)** describe the results for bacterial β-diversity; **(D,E)** describe the results for fungal β-diversity. **(A)** PCoA analysis of genera showed the relative distances between samples in different stages of dietary change; each pattern represents a different individual in a different stage, so the individual differences are also reflected in the relative distances. **(B)** and **(D)** are based on an Adonis analysis; “Between” represents differences between sample groups (according to sampling time), and the other bars, named “s (sample) + year (last two digits) + month (two digits),” represent the individual differences at each sampling time. *Y*-axis scale represents rank of distance. **(C,E)** Hierarchical clustering tree at the genus level. The lengths between branches represent the distances between samples marked by (Age + name); in **(C)**, sampling time points in S1 and S2 are colored blue, those in S3 are colored purple, and those in S4 are colored green; in **(E)**, sampling time points in S1 and S2 are colored blue and those in S3 and S4 are colored purple.

A principal coordinates analysis (PCoA) and Adonis analysis based on the Bray–Curtis values showed that the gut bacterial components of both cubs varied significantly during the period of dietary change (*P* < 0.05) and that the differences among the different sampling times were considerably greater than the differences between cubs (Figures 2A,B and Supplementary Figure S2). In particular, according to PC1, the bacterial structure after 2 years of age was clearly different (59.91%) from that in other periods, and the distant clustering of the gut bacterial components of the two panda cubs before and after the age of 2 years suggests that the bacterial community of the giant panda is restructured after 2 years of age. There were also large differences in the bacterial components of the two cubs also when they were aged 1.5–2 years, as indicated by the distant hierarchical clustering based on the Bray–Curtis analysis (group average) (Figure 2C).

We also investigated the fungal diversity [based on partial internal transcribed spacer 1 (ITS) sequences] in the two giant panda cubs. Overall, the fungal abundance, reflected in S_obs_ and the Chao index, increased in both cubs overall during the period of dietary change, as shown in Supplementary Figure S3, but some fluctuations were observed around 1.5 years of age (*P* < 0.05 on Welch’s *t*-test; Supplementary Figure S4). The fungal Shannon’s index of the giant panda cubs also varied around 1.5 years of age, and a decreasing trend indicated reduced heterogeneity in the fungal community (*P* < 0.05 on Welch’s *t-*test; Supplementary Figure S4), which was exactly opposite the variation detected with S_obs_ and Simpson’s index (Supplementary Figure S3). The fungal Shannon’s and Simpson’s indices gradually recovered after 2 years of age. We compared the fungal diversity in the two individuals, which did not differ significantly when the cubs ate a stable formula diet.

However, large difference of fungal components among all the samples from the different sampling periods were detected with PCoA and Adonis analyses based on the Bray–Curtis dissimilarity values (*P* < 0.05; Figure 2D and Supplementary Figure S5). The distances in the hierarchical clustering based on the Bray–Curtis values (group average) were greatest around 1 and 1.5 years of age (Figure 2E). This indicates that the fungal population structure of the two cubs varied continuously until they were 2 years old, which is consistent with the findings of the fungal α-diversity analysis. The gut fungal components of the giant panda cubs were similar during the periods when they started to eat bamboo and when they mainly ate bamboo, as shown in Figure 2E.

### Succession of Gut Microbial Composition and Structure in Giant Panda Cubs During Dietary Change

**FIGURE 3:**
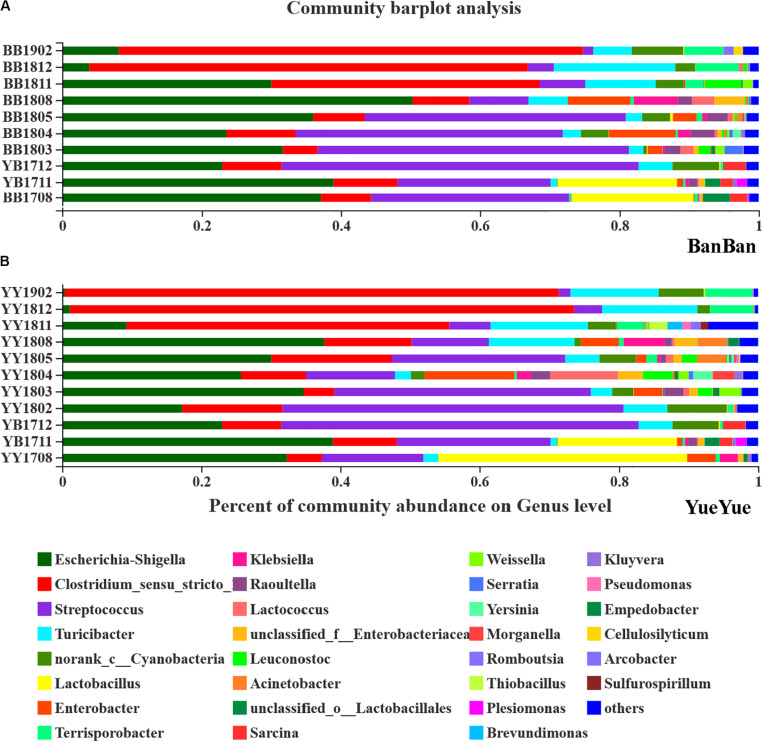
Bar-plot analysis of bacterial communities in panda cubs during their growth and dietary changes. **(A,B)** Bar-plot analysis of bacterial communities in BanBan and YueYue (other non-dominant taxa, with abundance <1%, were combined), respectively. Different colors represent different taxa at the genus level, and lengths are proportional to the abundance of the genus.

After each sample was analyzed with homogenization to an equal sequencing depth (50,427 effective reads per bacterial sample and 54,767 effective reads per fungal sample) and clustered, 844 and 1972 OTUs (at 97% identity) were identified, respectively. At the bacterial phylum level in both cubs, Firmicutes and Proteobacteria were the main phyla in almost 95% of all samples, followed by Bacteroidetes and Actinobacteria (Supplementary Figure S6). Phylogenetic maximum-likelihood (ML) trees showed that *Clostridium_*sensu_stricto_1, *Streptococcus*, *Turicibacter*, *Lactobacillus*, and *Terrisporobacter*, which belong to Firmicutes, and *Escherichia–Shigella*, which belongs to Proteobacteria, were the dominant genera in the giant panda cubs (Figure 3 and Supplementary Figure S7).

*Lactobacillus* and *Escherichia–Shigella* were two of the largest populations when the cubs still mainly consumed milk. After the cubs were 1 year old, with bamboo-adaptive training, the percentage of *Streptococcus* clearly increased and that of *Lactobacillus* decreased. *Streptococcus* and *Escherichia–Shigella* were the two largest taxa at the genus level, and other species, such as *Turicibacter*, *Raoultella*, and *Enterobacter*, gradually increased when the food components became complex. When the two individual cubs were compared, the dominant bacterial components were similar but their percentages were complex and varied, with individual differences at around 1.5–2 years of age (Figure 3).

After the cubs were predominantly consuming bamboo, with no milk, especially at >2 years old, *Clostridium* accumulated in large quantities, reaching 73% on the high-polysaccharide diet, which might explain the variations in the gut bacterial diversity, as well as the proportions of *Terrisporobacter* and other non-dominant taxa (<0.01) that increased in the giant panda on a bamboo diet (Figure 3). The dominant *Escherichia–Shigella* and *Streptococcus* declined after the cubs reached 2 years old and consumed only bamboo (Figure 3). Other genera, such as *Enterobacter* and *Raoultella*, whose populations increased when complex food was consumed, also decreased after the giant pandas moved onto a mainly bamboo diet. Bacterial dominance was very sensitive to variations in the cubs’ diet changed from milk to bamboo, and *Escherichia–Shigella* and *Clostridium* were particularly affected.

**FIGURE 4:**
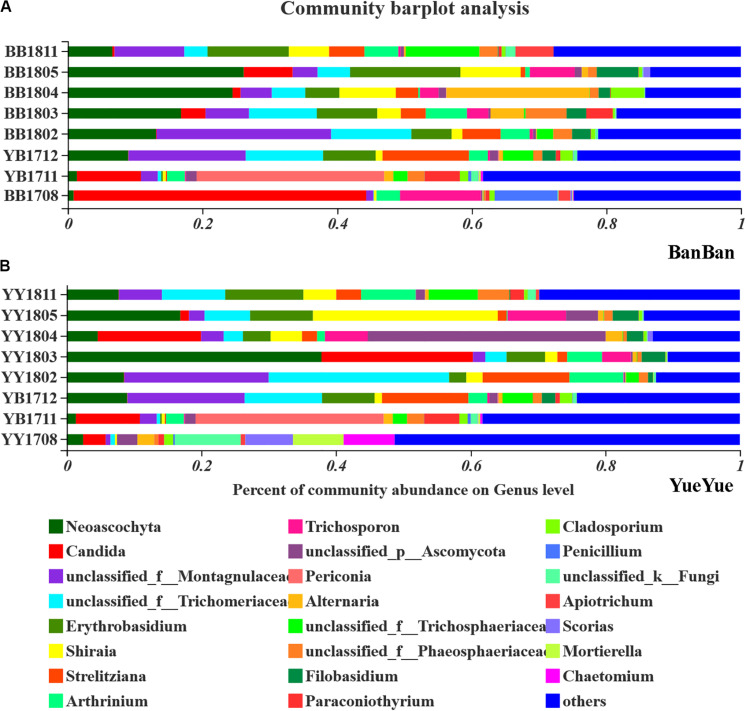
Bar-plot analysis of fungal communities in panda cubs during their growth and dietary changes. **(A,B)** Bar-plot analysis of fungal communities in BanBan and YueYue (others non-dominant taxa, with abundance <5%, were combined), respectively. Different colors represent different taxa at the genus level, and lengths are proportional to the abundance of the genus.

The gut fungi of both pandas were dominated by Ascomycota and Basidiomycota at the phylum level when analyzed with ITS1 sequences (Supplementary Figure S8). According to the phylogenetic (ML) trees, *Shirala*, unclassified_Trichomeriaceae, *Neoascochyta*, and unclassified_Montagulaceae in the phylum Ascomycota and *Erythrobasidium* in the phylum to Basidiomycota were the dominant genera (Figure 4 and Supplementary Figure S9). The five dominant fungi at the genus level accounted for a stable total proportion of > 50% in the two cubs after 1 year of age, as supplementary food and bamboo increased. The proportions of *Shirala*, *Neoascochyta*, unclassified_Montagulaceae, and unclassified_Trichomeriaceae varied significantly (*P* < 0.05), but not according to any regular characteristic or food preference of the cubs until they were 2 years old. At that time, the dominant populations were evenly distributed again, with no obviously dominant taxon in the gut (Figure 4 and Supplementary Figure S10). This is consistent with the variations in fugal diversity. These results demonstrated that the bacterial population showed a stronger and clearer response to dietary change than the fungal population.

### Gut Microbial Functions in Polysaccharide Metabolism of Giant Panda During Dietary Change

We also analyzed the functions of the gut bacteria and fungi by predicting the genes that were mainly involved in the polysaccharide metabolism of the giant panda cubs. Enzymatic activities were predicted with PICRUSt 2 based on KEGG database. Endoglucanase (EC 3.2.1.4) was associated with cellulose degradation in the gut bacterial genome. The frequency of predicted genes encoding endoglucanase (EC 3.2.1.4) fluctuated during the dietary shift process, but with a greater frequency than was observed in fungi. Unexpectedly, the predicted genes of putative microbial cellulases had existed in microbiome when the pandas ate supplementary foods, such as bamboo shoots (in S1) and coarse pastry (in S2).

**FIGURE 5:**
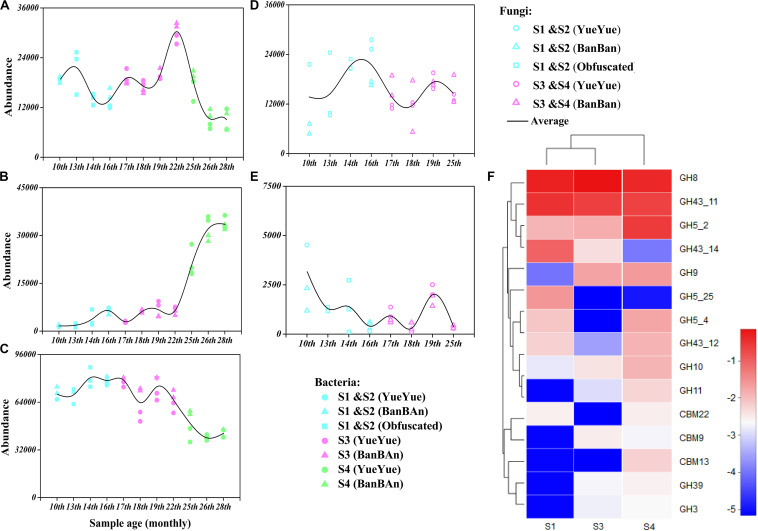
Genes encoding polysaccharide (starch, hemicellulose, and cellulose)-metabolizing enzymes were analyzed with functional prediction and metagenomic sequencing. Bacterial genes mainly encoding the polysaccharide-metabolizing enzymes cellulase [EC 3.2.1.4 **(A)**], hemicellulase [EC3.2.1.37 **(B)**], and α-amylase [EC3.2.1.1 **(C)**] were inferred based on 16S rRNA sequences and were predicted with PICRUSt 2, and the fungal genes encoding the polysaccharide-metabolizing enzymes cellulase [EC 3.2.1.4 **(D)**] and hemicellulase [EC3.2.1.8 **(E)**] were inferred based on ITS sequences and predicted with PICRUSt 2. Colored points represent samples from different stages of dietary change. Blue points represent samples from stage 1 and stage 2 (formula and bamboo chewing), purple points represent samples from stage 3 (increasing bamboo consumption), and green points represent samples from stage 4 (bamboo diet). Black trend lines indicate the average values for the two cubs and show the changes in the abundances of functional genes. **(F)** Heatmap of the dominant GHs and CBMs that correspond to cellulase (EC 3.2.1.4) and hemicellulase (EC 3.2.1.8 and 3.2.1.37), based on a metagenomic sequencing analysis and annotated with the CAZy database. Deeper red color indicates higher abundance of the gene; deeper blue color indicates lower abundance of the gene.

We predicted that the frequency of genes encoding endo1,4-β-xylanase (EC3.2.1.8), a key enzyme in hemicellulose degradation would be at least 10 times less in the giant panda cubs than that of endoglucanase (EC 3.2.1.4). Only β-D-xylosidase (EC 3.2.1.37), α-D-glucuronidase (EC 3.2.1.55), and ferulic acid esterase (EC 3.1.1.73) act in the hemicellulose-based hydrolase system (Zhang et al., 2019), and among these, genes encoding β-D-xylosidase (EC 3.2.1.37), a secondary key enzyme in hemicellulose degradation, was with more frequency in the cubs’ gut bacteria. The frequency of α-amylase (EC 3.2.1.1) gene was four times more than the cellulases gene in the panda microbiome (Figure 5 and Supplementary Tables S3, S4).

Because the functional gene predictions were based on amplified 16S rRNA and ITS microbial sequences and functional genera, they might have overestimated the actual distribution of functional genes in microbial genome. Therefore, we used metagenomic sequencing to verify the predicted activities of polysaccharide-metabolizing enzymes in the gut microbiomes of the giant panda cubs. Raw data (>10 Gb) were obtained from each sample. After the data were filtered and assembled, the average number of contigs per sample was 195,644, the average genome size was 150,702,282 bp, and the average N50 was 1,208 bp. The sequences were merged with ORF prediction and any redundancy was removed. The number of genes in the NR gene set was 724,019, with a total length of 358,038,903 bp. All NR genes were annotated in KEGG and CAZy databases.

The gut microbial genomes of the giant panda cubs already included endoglucanase (EC3.2.1.4) genes before they began to consume bamboo (S1), corresponding to GH members of GH5, GH5_25, and GH74, and their abundance increased slightly in general after the panda cubs ate a bamboo diet (S3 and S4), corresponding to GH5_2, GH8, and GH9. The abundance of β-glucosidase (EC 3.2.1.21) genes was also high in the panda cubs, corresponding to GH1 and GH3, but no cellobiohydrolase (EC3.2.1.91) genes were detected. However, genes encoding GH94, a cellobiose phosphorylase, were abundant in the gut microbial genomes; thus, microbes might have been present that could produce cellobiose with other types of exocellulases (Figure 5 and Supplementary Tables S3, S4). A metabolic pathway for cellulose degradation can be achieved with these enzymes mentioned above (Supplementary Figure S11).

The endo1,4-β-xylanase (EC3.2.1.8) genes were rare in the gut microbial genome, and only genes encoding β-D-xylosidase (EC 3.2.1.37) and α-D-glucuronidase (EC 3.2.1.55) were involved in hemicellulose degradation. The corresponding CAZy GHs were GH5, GH8, GH10, GH30, GH43, and GH51. However, the more abundant genes encoding β-D-xylosidase (EC 3.2.1.37) reduced in S3 and S4, with lower abundances than cellulase genes. The α-amylase (EC3.2.1.1) genes, corresponding in CAZy to GH13 and its subfamily, were about four times more abundant than genes annotated to cellulases and hemicellulases, but gradually decreased with bamboo consumption (bamboo:coarse pastry = 20:1, 0% starch in the bamboo and 41% in the coarse pastry; Table 1, Figure 5, and Supplementary Tables S5, S6).

These results suggested that microbial cellulose metabolism has more active potential than microbial hemicellulose metabolism, especially in bacteria, which were better able than fungi to provide abundant cellulose-metabolizing genes when the panda cubs increased their consumption of bamboo. The abundance of microbial amylase genes decreased as the proportion of starch in the cubs’ food decreased; thus, it was not a potentially useful polysaccharide metabolized by gut microbes for panda, although it could be directly metabolized by the panda itself.

### Identification of the Contributors of Genes Involved in Cellulose and Hemicellulose Metabolism in Giant Panda Cubs

We analyzed the correlations between almost all the functional bacterial populations in the giant panda cubs and the polysaccharide-metabolizing genes in the gut microbial genome. Proteobacteria and Firmicutes accounted for the largest proportions of the microbial population associated with polysaccharide metabolism, together including more than 44 genera and 112 species. Among these, *Enterobacter, Escherichia*, and *Paenibacillus* were the predominant genera participating in cellulose metabolism (Supplementary Figure S12 and Supplementary Tables S7, S8).

*Escherichia* (*E. coli*), *Shigella* (*S. sonnei*), *Enterobacter* (unclassified_f_Enterobacteriaceae), and *Clostridium* (*C. butyricum*) were the predominant species providing endoglucanase (EC.3.2.1.4) genes in the panda cub guts in S1. *Clostridium* (*C. butyricum*) and *Enterobacter* (such as unclassified_f_Enterobacteriaceae) were the dominant contributors to cellulose-metabolizing genes, corresponding to GH members GH5_25 and GH8, together with *Enterobacter* (*Ent. asburiae*), which was both a predominant species in the bacterial community and a major contributor to endoglucanase genes. When the panda cubs increased their consumption of bamboo in S3 and S4, *Escherichia* (*E. coli*) and *Enterobacter* (unclassified_f_Enterobacteriaceae) were the dominant contributors to cellulose-metabolizing genes, together with *Paenibacillus* (*P. elgii*) and *Klebsiella* (*K. variicola*) in S3 and *Lachnoclostridium* (*L. phytofermentans*) and *Escherichia* (*Escherichia*. sp. TW09276) in S4, corresponding to GH members GH5_2, GH8, and GH9. However, although most of these taxa were active potential contributors to cellulose metabolism, they were not predominant species in the panda cubs, so cellulose metabolism was probably still inefficient when the cubs consumed a complex diet, and the bacterial cellulose-metabolizing structure was unstable. The dominant populations mainly provided GH8. Other putative cellulases genes, such as those encoding GH5, were ascribed to *Cellulosilyticum*, *Lachnoclostridium*, *Clostridium*, and *Paenibacillus* and present at low levels; GH9 was contributed by *Klebsiella*, mainly in S3 and S4 (Supplementary Table S7).

Although GH5, GH8, GH43, GH51, GH10, GH11, and GH30 are annotated as endo1,4-β-xylanase (EC 3.2.1.8) in the CAZy database, the endo1,4-β-xylanase genes in the gut microbial genomes of the panda cubs only corresponded to genes of GH10 and GH11, which were provided by *Clostridium* (*C. disporicum*) and *Lactococcus* (*L. lactis*) and present at low levels. Moreover, the abundance of genes encoding β-D-xylosidase (EC 3.2.1.37), which corresponded to GH43_11 and GH43_12 and was mainly contributed by *C. disporicum*, *L. lactis* and *Lactobacillus* (*Lac. mucosae*), and *Streptococcus* (*Str. pasteurianus*), decreased on a bamboo diet (Supplementary Tables S7, S8).

**FIGURE 6:**
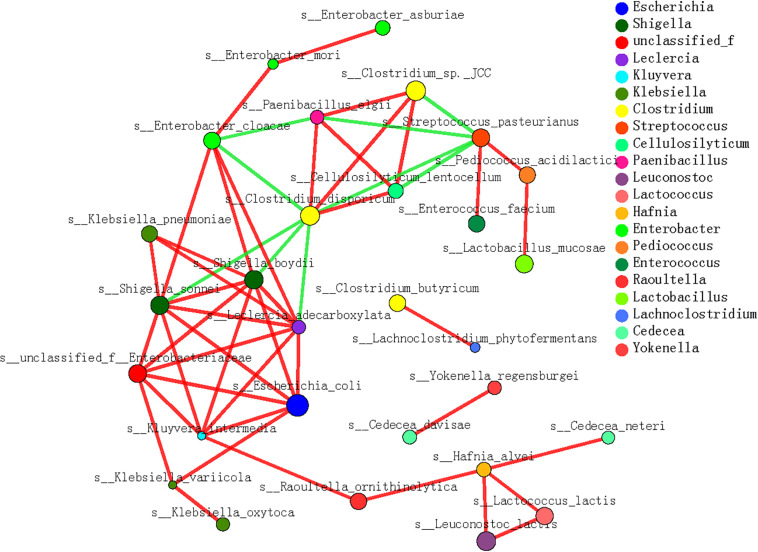
Microbial interaction grid for cellulose and hemicellulose degradation based on Spearman’s correlation analysis. Almost all the dominant bacterial genera (species) involved in cellulose and hemicellulose degradation are included in this grid; the same species have the same color. The sizes of the nodes represent the abundance of the species: the greater the abundance, the larger the node. The color of the connecting line indicates a positive or negative correlation: red indicates a positive correlation between species and functions, and green indicates a negative correlation between species and functions. The thickness of the line indicates the magnitude of the correlation coefficient: a thicker line indicates a stronger correlation. The more lines, the closer the relationships between this species/function and other species/functions.

In the microbe correlation grid based on the Spearman’s correlation analysis, almost all the dominant contributors to cellulose- and hemicellulose-metabolizing genes were involved with diverse interactions (Figure 6). Generally, positive correlations between species were more frequently detected during the dietary shift process. In particular, the interactions among different microbial species were more complex and stronger when the pandas ate a complex diet and increased their bamboo consumption (S3 and S4) than in the earlier stages. These included interactions between *P. elgii* and *Cellulosilyticum* (*C. lentocellum*) and between *Enterobacter* (unclassified_f_Enterobacteriaceae) and *K. variicola*. Moreover, when the giant panda cubs ate a bamboo diet, potentially cellulose-metabolizing species correlated positively with hemicellulose-metabolizing species, such as *P. elgii* and *Clostridium* (*Clostridium* sp. JCC), and *Leuconostoc* (*L. lactis*) and *Hafnia* (*H. alvei*).

Therefore, cellulose was hopefully the main bamboo polysaccharide used by the cubs, considering the complex metabolizing strategies of the gut microbes. The potentially cellulose-metabolizing bacterial species and their functional genes changed as the cubs’ diet changed, and microbial interactions were positive to deal with complex lignocellulose in bamboo.

## Discussion

### Succession of Gut Microbial Diversity and Structure During Bamboo Adaptation by Giant Panda Cubs

In this study, we monitored twin panda cubs living together; to avoid individual differences, we did not design food-controlled feeding trials (with different individuals) but demonstrated the diversity and predominant taxa in the intestinal microbial communities in twin cubs by evenly sampling every 1–3 months from 10 to 28 months of age during the dietary changes. The gut microbial population structure clearly varied in that period and allowed the pandas’ growth to be divided into three stages: 10 months to 1.5 years old, 1.5–2 years old, and after 2 years old. Although the panda cubs were trained to eat bamboo after 1 year of age, they mainly played with biting it first. The increase in bacterial and fungal richness in the two pandas were associated with food supplementation (Kieckhven and Wolf, 2016; Stephens et al., 2016; Williams et al., 2018; Guevarra et al., 2019). We continuously monitored the marked fluctuation of bacterial diversity and relative abundances after the pandas were 1.5 years old and the bacterial community stabilized when pandas had adapted to a specialized bamboo diet after they were 2 years old, differing from those of Zhang et al. (2018). That research group has reported recently that the intestinal microbial structure of giant panda cubs stabilized after they were 1 year old by investigating the fecal microbes in different individuals on bamboo diet or not. Therefore, we infer that the intestinal microbial structure is closely related to the composition of the diet, and is not simply dependent on the animal’s age.

At the phylum level, the dominant bacteria in the giant panda gut were Firmicutes and Proteobacteria, and the dominant fungi were Ascomycota and Basidiomycota (Guo et al., 2019). Among them, Bacteroides, a phylum that is a major source of polysaccharide-metabolizing bacterial taxa (McNulty et al., 2013; Zhu et al., 2018), but is scarce in the intestine of panda. Previous studies have shown that many members of Bacteroides could effectively degrade lignocellulose in the herbivore intestine not in the carnivore intestine (Yang et al., 2018). They may also be more adaptable to consume dietary fiber, such as grain, rather than the complex lignocellulose found in bamboo (McNulty et al., 2013). We will focus on this question in future studies. *Enterobacter*, *Escherichia–Shigella*, and *Lactobacillus* were the dominant bacterial genera in early life (Zhang et al., 2018). These populations prefer to colonize carnivore intestines and subsist on oligosaccharides, fats, amino acids, and their derivatives in cubs’ food (Varrone et al., 2013; Kong et al., 2018; Li et al., 2019), and are associated with many important energy metabolism pathways (Guo M. et al., 2018); in particular, *Lactobacillus* is a probiotic genus in early growth of mammals (Williams et al., 2013). The relative proportion of *Streptococcus* increased in the cub guts after the age of 1 year when consuming the products of fibrous metabolism (Xue et al., 2015; Wang et al., 2016) in supplementary foods. However, the predominant *Escherichia–Shigella* and *Streptococcus* decreased as the low-energy-consumption members continued to increase and on which the high cellulose in bamboo had less impact, including *Clostridium* and *Turicibacter* (Williams et al., 2013; Song et al., 2017; Tang et al., 2019) especially after the pandas were 2 years old. Therefore, we infer that forming an energy-saving dominant microbial structure may be one of the most important strategies for panda and their microbes to adapt to a bamboo diet at this time.

Both cubs had similar fungal diversity after they began to consume bamboo, and the proportions of the dominant taxa remained large between 1.5 and 2 years old. Therefore, fungi are probably ingested into the gut and are influenced by dietary change. *Shirala* is only present in bamboo, as Lin et al. (2008) reported that *Shirala* preferentially lives on bamboo and proliferates in spring more than in the other seasons, and our research found that the relative proportion of *Shirala* were greatest in spring (May 2018). The other dominant genera, such as *Neoascochyta* and *Erythrobasidium*, were also generally from plant sources (Wang and Bai, 2004). Unlike the fluctuations in bacterial diversity and richness, the fungal population structure in the 2-year-old giant pandas on a bamboo diet was very similar to that observed when they first began to consume bamboo, with no obvious predominant taxa. The food components in both periods were relatively simple. This stability may be a protective mechanism to control the proportions of dominant fungi present in a nutrient-poor intestine, to prevent pathogen proliferation. Whether fungi positively or negatively affect the use of bamboo by the giant panda in the complex dietary stage warrants further investigation. However, we found no obvious association between the succession of dominant fungi in the giant panda cubs and their adaptation to nutrient utilization (Gonzalo et al., 2016; Zhang et al., 2018). Fungi are the main lignocellulose degraders (Brink and Vries, 2011). *Aspergillus*, *Penicillium*, and *Trichoderma* are genera that effectively break down lignocellulosic composites (Carvalho et al., 2013; Gaurav et al., 2017), and white-rot fungi are equipped with a rich suite of extracellular oxidative enzymes to attack and degrade lignin better than bacteria (Gonzalo et al., 2016). However, these fungal taxa were rarely present in the giant panda cubs’ guts.

### Contribution of Gut Bacteria to Polysaccharide Metabolism by Providing Functional Genes in Giant Panda Cubs

The nutritional composition of the cubs’ food varied during the period of dietary change. Polysaccharides were present in large amounts, stored in bamboo as cellulose (40–50%) and hemicellulose (10–30%). According to our functional gene prediction and metagenomic sequence analysis, the potentially microbial metabolism related to cellulose degradation was more common than hemicellulose degradation in the giant panda cubs during their dietary shift. The endoglucanase (EC 3.2.1.4) and β-glucosidase (EC 3.2.1.21) genes generally increased as their hosts increase bamboo consumption. These are the key enzymes in the metabolic pathway of cellulose degradation and correspond to CAZy members GH1, GH3, GH5, GH8, and GH9 [The carbohydrate-active enzyme (CAZy), 2020]. Although we detected no cellobiosidase (EC3.2.1.91) genes in the twin panda cubs (Yang et al., 2018), genes encoding cellobiose phosphorylase (GH94) increased in the gut microbial genome. Therefore, the capacity of the giant panda to degrade the cellulose in bamboo is potentially active, attributed to the enzyme gene groups of its gut microbes.

Actually, we only detected the GH10 gene in the two panda cubs, encoding endo1,4-β-xylanase (EC 3.2.1.8), with more than 10 times fewer genes than endoglucanase (EC3.2.1.4), and the GH43 subfamily genes, encoding xylan 1,4-beta-xylosidase (EC3.2.1.37), and these genes decreased with increased bamboo consumption. Zhang et al. (2018), have revealed that the genes encoding GH1, GH4, GH8, and GH31 were involved in hemicellulose degradation. However, based on different individuals, with different sampling, and under different experimental conditions and analytical methods, these differences can seriously affect the gut bacterial genome, which is addressed in the rationale of our experimental design.

Considering that bacteria could provide more polysaccharide-metabolizing genes than fungi, and no potential polysaccharide-degrading fungi were detected in the giant panda cubs, as discussed above, we infer that bacteria are the most important contributors to the metabolism of the polysaccharides in bamboo. Interestingly, the type and abundance of cellulose-metabolizing genes in the pandas varied with the dietary shift, and the corresponding members of the GHs were provided by different bacterial species (Zhu et al., 2011; Guo W. et al., 2018). GH5_25 genes mainly appeared in the gut bacterial genome of the panda cubs before they began to consume a bamboo diet, and it was provided by *C. butyricum*. *Bifidobacterium* was also detected at that time, providing GH5_4 genes. Both of them may degrade fermentable fiber substrates in the panda (Williams et al., 2013; Xia et al., 2014) by expressing GH5 (Wierzbicka-Wo et al., 2019). When the cubs consumed supplementary foods and bamboo, only GH5_2 genes produced by *Paenibacillus* was still abundant in the gut microbes, which is a special GH5 subfamily with high activity and stable structure (Ma et al., 2020). Enterobacteriaceae, *Escherichia*, and other taxa increased genes encoding GH8 and GH9. It is unexpected that the *E. coli* providing GH8 may become a main contributor of putative cellulase (EC 3.2.1.4) in cubs. Actually, as generally considered, the *E. coli* cannot degrade cellulose, and more than 75% of the endoglucanases from GH8 produced by bacterial taxa such as *E. coli* at Proteobacteria are as a component of the bacterial cellulose synthesis (bcs) system (Berlemont and Martiny, 2013), but interestingly, most of them are potentially required to correct packing of cellulose microfibrils (Mazur and Zimmer, 2011); that is, GH8 may retain its activity of endoglucanase to cello-oligosaccharides in the bcs system (Scapin et al., 2017). Under the product enrichment conditions, such as microfibril-like structured cellulose, GH8 will be secreted extracellularly, and out of the bcs system, it can hydrolyze soluble cellulose and cello-oligosaccharides in the environment (Pang et al., 2019), and structure-specific GH8 subfamily can directly hydrolyze amorphous CMC and crystalline cellulose (Attigani et al., 2016). In this special environment with large amount of bamboo fiber and complex microbial interaction, how the bacterial endoglucanases from GH8 participate in cellulose metabolism is attractive for further research. GH9 is more specific for complex cellulose polymer degradation (Vita et al., 2019), potentially coupled with other GHs such as GH5, GH8, and GH48 in cellulosomes for the degradation of complex lignocellulose (Xu et al., 2013).

However, only those contributors that also provide genes encoding β-glucosidase (GH1 or GH3) can be the potential cellulose degraders (Berlemont and Martiny, 2013). The dominant potential cellulose degrader in panda included *C. butyricum* (providing GH1 and GH5 genes), whereas species such as *Bifidobacterium* and *Paenibacillus* only contained GH5 genes, so they might not degrade cellulose completely and relied on other microorganisms. Other dominant populations such as unclassified_f_Enterobacteriaceae and *Ent. Asburiae* contained GH8 and GH1 genes; however, whether they can be the cellulose degraders rely on the function of GH8 as mentioned above. There were also some non-dominant species that could be regarded as potential cellulose degraders by providing GH1, GH3, GH5, and GH9 genes, such as *Cedecea* (*Ced. davisae* and *Ced. neteri*) and *Klebsiella* (*K. pneumonia and K.* variicola), when panda cubs ate bamboo diet. In general, as the succession of contributors and potential degraders related to cellulose metabolism, the enzyme gene group for bamboo cellulose metabolism may be gradually developed in gut microbiome of giant panda. Those genes encoding endoglucanase (EC3.2.1.4) were mainly provided by species in Clostridiaceae, Enterobacteriaceae, Lachnospiraceae, and Paenibacillaceae, and genes encoding β-glucosidase (EC 3.2.1.21) were mainly provided by species in Enterobacteriaceae, Clostridiaceae, Lachnospiraceae, and Erysipelotrichaceae. Combining the correlation between cellulose metabolism pathway and potentially functional species, we consider that few species providing genes of endoglucanase such as GH5 and GH9 may be a main limiting factor for cellulose digestion in panda, which also need further exploration. However, we found that there were fewer and simpler species related to hemicellulose metabolism in the panda cubs, and they were mainly included in *C. disporicum* and *L. lactis.*

Actually, the potential for degrading the cellulose and hemicellulose in bamboo are both weak in the giant panda. As for the actual abilities of pandas to degrade bamboo polysaccharides, further analysis needs to be combined with expression of functional gene, substrate digestibility, and so on. Other food components, such as proteins, amino acid, starches, and other saccharides, are also used by the panda, but are not strictly digested by the gut microbes. For instance, starch consumption increased during the growth of the cubs, whereas the number of microbial α-amylase genes declined with the reducing proportion of starch in food. Large proportions of lignocellulose in food might potentially weaken the α-amylase activity of microbes; besides, microbial glycoside hydrolase is usually active in intracellular metabolism (Manohar et al., 2017; Chen et al., 2019). Therefore, absorbing those nutrients, such as starch, proteins, amino acids, and other saccharides, that occur in low relative proportions in the panda cubs’ bamboo diet may mainly rely on host enzymes, although we did not investigate these enzymes in this study.

Regardless of how difficult it is for the panda to degrade the lignocellulose in a bamboo diet, as described above, microbial interactions correlated positively with cellulose metabolism and the dietary shift. In this study, we detected strong positive correlations between functional species related to cellulose metabolism. Furthermore, the interactions among different species were more complex and stronger when the pandas consumed a complex diet that included bamboo and supplementary foods (Makki et al., 2018; ZinÖcker and Lindseth, 2018). Moreover, when the giant panda cubs consumed a bamboo diet, positive interactions were detected between specific species related to cellulose- and hemicellulose-metabolizing, such as *P. elgii* and *Clostridium* sp. JCC, and *L. lactis* and *H. alvei*. Therefore, the distribution and activation of polysaccharide-metabolizing genes in the gut microbiome also rely on the microbial interactions in the panda gut.

## Conclusion

Based on the results reported above, we conclude that (i) the gut microbial structure changes during the dietary shift, and that the dominant microbial community develops in giant panda cubs after the age of 2 years; (ii) gut bacteria are the main sources of cellulolytic genes in giant panda cubs, whereas hemicellulose is used negligibly by these gut microbes; (iii) cellulose degradation relies on complex metabolizing strategies: the types and abundances of cellulose-metabolizing species and functional genes vary with dietary shifts, and the potential cellulose degraders belong to *Clostridium*, *Cedecea*, and *Klebsiella*, providing GH1, 3, 5, and 9 genes; gut microbial interactions also have a very positive link with polysaccharide degradation. In general, those functional species are not the predominant taxa in the panda cub, so utilization of bamboo polysaccharides may still be weak in the panda. Of course, it will be necessary to further explore the actual expression of the functional microbial genes and to explain the microbial mechanism of bamboo cellulose metabolism such as GH8 in the panda.

## Data Availability Statement

The datasets generated for this study can be found in the NCBI, PRJNA626529.

## Author Contributions

MZ, LW, CXi, XF, SZ, AW, YZ, CXu, and HZ performed the material preparation, data collection, and analysis. MZ wrote the first draft of the manuscript. LW revised the manuscript critically. All authors contributed to the study conception and design, commented on previous versions of the manuscript, and read and approved the final manuscript.

## Conflict of Interest

CXi and CXu were employed by the company Shanghai Wild Animal Park Development Co., Ltd. The remaining authors declare that the research was conducted in the absence of any commercial or financial relationships that could be construed as a potential conflict of interest.

## Footnotes

1 https://ccb.jhu.edu/software/FLASH/index.shtml

2 http://drive5.com/uparse/

3 www.illumina.com

4 www.majorbio.com

5 https://github.com/OpenGene/fastp

6 https://github.com/voutcn/megahit

7 http://metagene.cb.k.u-tokyo.ac.jp/

8 http://www.bioinformatics.org/cd-hit/

9 http://soap.genomics.org.cn/

10 http://www.diamondsearch.org/index.php

11 http://hmmer.janelia.org/search/hmmscan

## Supplementary Material

Supplementary Data Sheet 2 The KEGG and CAZy annotations of gut functional microbes in giant panda.

Supplementary Data Sheet 3 Overview at CAZy family of gut microbes in giant panda.
