# Supplementary material for: Succession of Gut Microbial Structure in Twin Giant Pandas During the Dietary Change Stage and Its Role in Polysaccharide Metabolism
Source: Front Microbiol. 2020 Sep 22;11:551038. doi: 10.3389/fmicb.2020.551038 (PMC7537565; doi:10.3389/fmicb.2020.551038)
Supplement: Supplementary file 1 [file Data_Sheet_1.PDF]

Supplementary Material

Succession of Gut Microbial Structure in Two Twin Giant Pandas during the Food Conversion Stage and Its Functions in Polysaccharide Metabolism

Mingye Zhan[1], Lei Wang[1, *], Chunyu Xie[2], Xiaohua Fu[1], Shu Zhang[3], Aishan Wang[3], Yingmin Zhou[4,*], Chunzhong Xu[2], Hemin Zhang[4]

[1] *College of Environmental Science and Engineering in Tongji University, Institute of Pollution Control and Ecological Safety, Shanghai 200092, China*

[2] *Shanghai Wild Animal Park Development Co., Ltd., Shanghai 201399, China*

[3] *Shanghai Zoo, Shanghai 200335, China*

[4] *Chinese Giant Panda Protection Research Center, Dujiangyan 611830, China*

[*] **Correspondence**

Lei Wang (e-mail: celwang@tongji.edu.cn)

Yingmin Zhou (email: 124156610@qq.com)

Includes:

Supplementary Methods

Supplementary Tables

Supplementary References

Supplementary Figures

Supplementary Methods

Sample collection

Sampling began in August 2017 and continued to February 2019 (once every 1–3 months, a total of 10 times): August, 2017; November, 2017; December, 2017; February, 2018; March, 2018; April, 2018; May, 2018; August, 2018; November, 2018; December, 2018 and February, 2019. Feces are extremely difficult to collect from panda ≤ 6-months-old. Fresh bamboo samples were collected and dried in an oven at no more than 80 ℃ to analyze their elemental contents.

DNA extraction

The DNA was extracted from the filter membranes with the PowerSoil® DNA Isolation Kit (Mo Bio Laboratories Inc., Carlsbad, CA, USA), according to the protocol of manufacturer. Each 0.5 g stool sample was inserted into the "Power Bead Tubes" and manually mixed. The C1 solution (dissolved at 60 ℃) was added, and the sample was vortexed for 10 min and centrifuged for 1 min at 13000 g. According to the protocol, the C2 and C3 solutions were added to the collected supernatant in turn, and the mixture incubated at 4 ℃ for 5 min with each addition. The collected supernatant was mixed with C4 solution and loaded onto a "Spain Filter". The DNA stayed bound to the filter membrane, and was washed with C5 solution, and then eluted and collected with ddH2O. The DNA concentration and quality were evaluated with a NanoDrop™ 2000 spectrophotometer (NanoDrop, Wilmington, DE, USA).

The bacterial DNA was amplified with the primers 338F (5'-ACTCCTACGGGAGGCAGCAG-3') and 806R (5'-GGACTACHVGGGTWTCTAAT-3'). Unique eight-base-sequence barcodes were attached to each sample. The PCRs were performed in triplicate in 20 μL reaction mixtures containing 4 μL of 5 × FastPfu Buffer, 2 μL of 2.5 mM dNTPs, 0.4 μL of each primer (10 μM), 0.4 μL of FastPfu Polymerase, 10 ng of template DNA, and ddH2O to the final volume. The amplicons were extracted from 2% agarose gels and purified with the AxyPrep DNA Gel Extraction Kit (Axygen Biosciences, Union City, CA, USA),

according to the manufacturer's instructions. The amplicons were quantified with QuantiFluor™-ST (Promega Corporation, Madison, WI, USA). Equimolar amounts of the purified amplicons were pooled and paired-end sequenced on an Illumina MiSeq 300 platform, with standard protocols. Fungi were amplified with the primers ITS1F (5'-CTTGGTCATTTAGAGGAAGTAA-3') and ITS2 (2043R) (5'-GCTGCGTTCTTCATCGATGC-3'). The PCRs were performed in triplicate in 20 μL reaction mixtures containing 2 μL of 10 × buffer, 2 μL of 2.5 mM dNTPs, 0.2 μL of rTaq polymerase, 0.4 μL of each primer (10 μM), 0.4 μL of FastPfu Polymerase, 10 ng of template DNA, and ddH2O to the final volume.

Analysis of polysaccharides contents of bamboo

After each sample was filtered through a 60-mesh sieve, 0.2 g was added to 60% sulfuric acid solution in a cold water bath and digested for 30 min. The mixture was made up to 100 mL with 60% sulfuric acid solution and filtered. In a colorimeter tube, 0.5 mL of 2% anthrone was added to the filtrate for anthrone colorimetry, and 5 mL of concentrated sulfuric acid was slowly added. After shaking, the cellulose content was determined with anthrone colorimetry method at 620 nm (Lu et al. 1996). The bamboo samples were sieved through a 60-mesh sieve and the lignin content was determined with redox titration according to Fan et al. (2008), with some minor modification. The starch was hydrolyzed with α-amylase. After the fat and soluble sugars were removed with organic solvents, the starch was hydrolyzed to disaccharides with amylase, which were then hydrolyzed to monosaccharide with hydrochloric acid. The starch content was converted to the amount of reducing sugar with enzymatic hydrolysis method (GB/T5009.9-2003, China)

Statistical Analysis

In this study, the data analysis of cellulose and hemicellulose in bamboo was performed with the RStudio-1.1442 software. One-way ANOVA was used to analyze the differences in the components of the bamboo that was the pandas' staple food in different seasons. All the data are expressed as means ± standard errors

(n = 3, P < 0.05). Cytoscape, as an open source software platform, was used for preforming the networks between specific cellulose degradation pathway in ko00500 (mainly including two key enzyme in the gut microbiome of giant pandas, Endoglucanase (EC3.2.1.4) corresponding to GH5_2 and GH5_25, and β-glucosidase (EC 3.2.1.21) corresponding to GH1 and GH3) and related dominant bacteria (> 25 species) in giant pandas with bamboo diet during S4.

Supplementary Tables

Supplementary Table S1: Results of basic information of experimental giant pandas.

Name	Lineage	Date of birth	Location	Health condition
YueYue	1052	Oct, 2016	Shanghai Wild Animal Park	Health
BanBan	1053	Oct, 2016	Shanghai Wild Animal Park	Health

Supplementary Table S2: The numerical values of α diversity indices of samples.

Sample information		α diversity index															
Sort	Age	Sobs				Chao				Shannon				Simpson			
		YueYue		BanBan		YueYue		BanBan		YueYue		BanBan		YueYue		BanBan	
Bacteria	10th	85		120	114	116.67		149.08	164.00	1.94		2.26	2.15	0.237		0.212	0.228
	13th	146		135	129	185		234.17	165.91	2.11		2.56	2.11	0.249		0.156	0.273
	14th	130		135	169	155.87		191.44	211.5	1.81		1.97	1.51	0.273		0.237	0.402
	16th	184	182	174	174	259.14	243.25	240.96	250.25	1.97	2.05	1.93	1.85	0.270	0.239	0.256	0.270
	17th	180	152	191	200	275.06	193.13	244.00	263.03	2.21	2.01	1.99	1.97	0.222	0.261	0.278	0.279
	18th	182	166	221	220	262.45	263.24	307.36	319.11	2.93	2.79	2.18	2.28	0.101	0.116	0.217	0.194
	19th	262	242	182	220	358.28	348.50	233.11	293.31	2.59	2.42	1.92	1.96	0.151	0.182	0.269	0.263
	22th	192	174	102	111	258.00	216.00	127.67	154.50	2.60	2.37	1.97	2.09	0.156	0.207	0.298	0.254
	25th	203	155	151	145	219.71	202.04	215.47	212.56	2.73	1.84	2.00	1.97	0.154	0.312	0.233	0.235
	26th	98	106	125	143	164.60	167.75	200.25	222.69	1.39	1.31	1.77	1.80	0.475	0.511	0.362	0.334
	28th	137	153	157	139	224.14	244.64	233.56	224.00	1.28	1.39	1.73	1.70	0.514	0.453	0.396	0.390
Fungi	10th	115		113	123	122.00		143.67	148.00	4.02		2.56	2.27	0.025		0.130	0.212
	13th	174		363		204.33		376		4.18		3.59		0.0274		0.0965	
	14th	317.5		302		352.25		330.99		3.51		3.69		0.0729		0.0633	
	16th	372	400	370	426	491.00	542.88	401.36	486.51	2.87	3.02	3.43	3.12	0.106	0.091	0.081	0.115
	17th	325	248	411	405	371.12	298.17	503.75	509.65	2.71	2.59	3.47	3.65	0.180	0.178	0.058	0.048
	18th	387	379	202	311	468.84	507.27	238.11	330.33	3.15	2.72	2.18	3.60	0.113	0.180	0.240	0.054
	19th	254	323	212	281	277.00	372.88	228.87	302.97	3.23	3.17	3.12	3.30	0.078	0.075	0.100	0.076
	25th	508	579	487	520	544.54	700.41	559.20	610.55	3.88	3.91	3.82	3.71	0.042	0.039	0.049	0.051

Supplementary Table S3: The abundance of polysaccharide-metabolizing enzymes analyzed by functional prediction (based on 16s and ITs sequencing) and metagenomic sequencing.

Method: 16s sequencing prediction																	
		EC 3.2.1.1				EC3.2.1.4				EC3.2.1.8				EC3.2.1.37			
Stage	Sample	Sample1	Sample2	Sample3	Sample4	Sample1	Sample2	Sample3	Sample4	Sample1	Sample2	Sample3	Sample4	Sample1	Sample2	Sample3	Sample4
S1&S2	S1708	65877.23	69918.81	74295.72		18664.51	19447.59	17946.48		584.64	130	74.79		1309.27	1979.61	1925.95	
	S1711	71067.23	62692.14	72668.87		23669.24	15101.47	25312.93		152.36	1008.09	6585.09		1203.17	2352.64	2017.13	
	S1712	78480.1	74419.06	87436.19		14405.6	15215.25	12600.14		4.67	34.33	59		2515.6	6805.08	2073.14	
	S1802	76806.57	75552.28	78981.11	81301.66	11910.63	12449.3	14315.89	16690.2	107.08	130.33	52.67	56.99	6525.83	7305.9	6809.51	5179.66
S3	S1803	74201.47	77624.9	80481.13	80385.8	18660.25	21386.77	17750.69	18168.64	13.42	13.33	42	51.25	3141.89	2687.63	2878.43	3104.17
	S1804	51212.96	57382.41	73212.57	71728.38	17609.12	18515.37	16178.2	15441.56	73	16	38.49	27	6205.81	5953.8	5711.3	6784.15
	S1805	65416.95	70051.93	80534.29	80371.94	18956.58	19416.54	21485.84	19082.7	484.28	280.6	16.33	82.67	8109.04	9376	4563.44	4733.24
	S1808	56853.81	63564.89	72160.63	67167.61	27323.01	29386.53	32342.19	31478.93	61.66	39.09	9	37.68	7594.43	7248.23	4980.56	6346.81
S4	S1811	37321.84	46409.71	55857.33	58075.78	18319.37	13474.61	19459.47	20858.27	3892.33	33	38	28.67	18040.66	27247.43	19990.46	18622.25
	S1812	40594.18	42305	38567.64	38584.01	7959.82	6919.15	9904.07	11599.69	16	25.57	24.27	44.42	34770.46	35936.62	30044.97	28192.59
	S1902	40529.66	45483.92	40728.09	45702.26	6727.56	11644.08	6670.68	10552.85	47.08	2866.65	89.83	2960.71	36328.56	32490.78	33452.25	31917.51

Method: ITs sequencing prediction																	
		EC 3.2.1.1				EC3.2.1.4				EC3.2.1.8				EC3.2.1.37			
Stage	Sample	Sample1	Sample2	Sample3	Sample4	Sample1	Sample2	Sample3	Sample4	Sample1	Sample2	Sample3	Sample4	Sample1	Sample2	Sample3	Sample4
S1&S2	S1708	130371.3	80604.3	72626.7		21601.5	4807	7182		4522.5	1184	2325		3328.5	606	1402	
	S1711	130546	119467.7	140709		24430	9761	9323		9096	1167	1359		6700	1393	1041	
	S1712	136861	127436.3	117905.9		20555	22872	20678		2728	117	1258		2319	24	250	
	S1802	130253.2	133565	123204	122263	25301	27531	16566	17415	209	153	598	519	52	63	383	376
S3&S4	S1803	131146	119570.7	137940	129640.7	11768	10975	14031.5	18858	875	1368	593.5	718	208	20	119.5	217
	S1804	83459.45	69961.45	165945	138544	12320	11694	5242	17675	155	103	234	582	126	92	269	608
	S1805	141872	138399	124814	121506	19534	15713	17363	16657	1997	2506	1948	1426	686	739	1721	1346
	S1811	124776.9	131316.7	132396	128847.7	12823	14354	12486	19018	284	326	441	293	1130	1105	1492	1019

Method: metagenomic sequencing								
	EC 3.2.1.1		EC3.2.1.4		EC3.2.1.8		EC3.2.1.37	
Stage	Sample1	Sample2	Sample1	Sample2	Sample1	Sample2	Sample1	Sample2
S1	12720	11670	3460	3692	158	98	7642	4052
S3	14840	25220	6182	4104	84	8	3462	1632
S4	4906	7684	2738	4626	24	254	722	3630

Supplementary Table S4: The corresponding GHs genes for cellulose-, hemicellulose- and starch-degrading annotated by CAZys with metagenomic sequences.

CAZy annotation	Abundance in sample						
	S1_1	S1_2	S2_1	S2_2	S3_1	S3_2	Total
CBM13	40	62	424	24	34	356	940
CBM16	98	394	412	160	1686	876	3626
CBM4	20	1874	188	94	384	1082	3642
CBM59	0	0	0	0	12	200	212
GH1	114290	89348	64398	91048	78910	89732	527726
GH10	6	86	196	122	186	280	876
GH11	0	0	6	12	10	26	54
GH3	13378	9720	10610	5804	7734	13658	60904
GH30	428	3126	6	0	0	0	3560
GH43	1296	6018	218	74	1046	1266	9918
GH43_11	1286	7186	2200	1204	442	2268	14586
GH43_12	34	394	48	26	40	478	1020
GH5	814	1534	602	1044	328	276	4598
GH5_2	8	230	230	72	1654	1712	3906
GH5_25	4	238	240	80	18	4	584
GH5_4	4	290	0	0	2	310	606
GH51	46	944	268	1090	1874	2688	6910
GH6	0	36	0	0	2	0	38
GH74	218	282	88	18	60	56	722
GH8	4114	7478	6028	3958	1044	2800	25422
GH9	14	20	256	360	316	220	1186
GH94	626	3480	1996	944	6588	6442	20076
CBM34	190	292	554	204	258	450	1948
GH13	4830	2110	2328	2444	4518	6692	22922
GH13_15	14	332	2	4	6	14	372
GH13_19	4418	3534	5958	4324	1190	2920	22344
GH13_28	8	460	112	664	0	0	1244
GH13_5	5878	6688	7236	18784	2280	3110	43976
GH14	2	4	96	50	178	92	422
GH15	258	184	460	148	106	134	1290
GH97	6	298	208	86	796	758	2152

Supplementary Table S5: Main polysaccharides content of bamboo in different season for giant panda.

	C（%）	N（%）	C/N（%）	Cellulose（%）	Lignin（%）	Starch (%)
Aug	45.01±1.78[a]	1.72±0.05[a]	26.13±0.26	29.75±2.76[b]	14.38±6.19	——
Nov	44.18±0.72	2.33±0.04[b]	19.26±0.29	32.51±4.01[a]	16.67±5.30	——
Feb	45.09±1.51	1.86±0.34	24.76±5.38	39.51±4.94[a]	15.21±7.37	——
May	40.68±0.06[b]	1.92±0.03	21.24±0.40	20.67±0.99[b]	15.63±3.24	——

Results expressed by mean ±standard error (n=3). Different letters meant significant difference between two samples (analysis in the same column) (P<0.05, n=3).

Supplementary Table S6: Complementary food of giant panda and nutritional composition (%) (Wu F et al. 2018).

Content	Coarse pastry		Apple		Carrot		Bamboo shoots	
	Summer and Autumn （n=5）	Winter and Spring （n=5）	Summer and Autumn （n=5）	Winter and Spring （n=5）	Summer and Autumn （n=5）	Winter and Spring （n=5）	Summer and Autumn （n=5）	Winter and Spring （n=5）
Moisture (%)	42.00±0.71	40.46±2.73	88.74±1.60	85.42±0.81	90.30±0.75	90.38±0.76	88.28±1.25	87.30±1.07
Crude protein (%)	10.81±0.24	11.92±0.63	0.24±0.05	0.27±0.02	0.77±0.10	0.67±0.06	1.86±0.15	3.04±0.15
Crude fat (%)	5.90±0.32	6.14±0.42	0.10±0.00	0.20±0.00	0.10±0.00	0.14±0.05	0.10±0.00	0.28±0.08
Calcium (%)	0.13±0.02	0.19±0.05	0.01±0.00	0.03±0.01	0.03±0.01	0.04±0.00	0.01±0.00	0.04±0.01
Total phosphorus (%)	0.30±0.11	0.21±0.02	0.01±0.01	0.00±0.00	0.03±0.01	0.02±0.00	0.04±0.01	0.04±0.00
Coarse ash (%)	1.88±0.08	2.00±0.16	0.50±0.19	0.28±0.06	0.88±0.16	0.50±0.08	0.76±0.05[a]	1.02±0.05[b]

Results expressed by mean ±standard error (n=3). Different letters meant significant difference between two samples (analysis in the same column) (P<0.05).

Supplementary Table S7 Functional bacterial species (genus) for polysaccharide degradation.

Genus	Species	KEGG_Enzyme	CAZY_Far
g__Clostridium	s__Clostridium_disporicum	3.2.1.8	GH10
g__Cedecea	s__Cedecea_davisae	3.2.1.4	GH8
g__Clostridium	s__Clostridium_disporicum	3.2.1.37	GH43_12
g__Enterobacter	s__Enterobacter_asburiae	3.2.1.4	GH8
g__Lactococcus	s__Lactococcus_lactis	3.2.1.37	GH43_11
g__Streptococcus	s__Streptococcus_pasteurianus	3.2.1.37	GH43_14
g__Kluyvera	s__Kluyvera_intermedia	3.2.1.4	GH8
g__Yersinia	s__Yersinia_mollaretii	3.2.1.4	GH8
g__Klebsiella	s__Klebsiella_pneumoniae	3.2.1.37	GH43_11
g__Enterobacter	s__Enterobacter_sp._DC1	3.2.1.4	GH8
g__Hordeum	s__Hordeum_vulgare	3.2.1.4	GH9
g__Klebsiella	s__Klebsiella_pneumoniae	3.2.1.4	GH8
g__Clostridium	s__Clostridium_longisporum	3.2.1.4	GH5_4
g__Hafnia	s__Hafnia_alvei	3.2.1.4	GH8
g__Cellulosilyticum	s__Cellulosilyticum_lentocellum	3.2.1.4	GH5_2
g__Cedecea	s__Cedecea_neteri	3.2.1.4	GH8
g__Clostridium	s__Clostridium_saccharoperbutylacetonicum	3.2.1.8	GH10
g__Enterobacter	s__Enterobacter_sp._638	3.2.1.4	GH8
g__Streptococcus	s__Streptococcus_pneumoniae	3.2.1.37	GH43_11
g__Enterobacter	s__Enterobacter_cloacae	3.2.1.4	GH8
g__unclassified_f__Enterobacteriaceae	s__unclassified_f__Enterobacteriaceae	3.2.1.4	GH8
g__Brachypodium	s__Brachypodium_distachyon	3.2.1.4	GH9
g__unclassified_f__Lachnospiraceae	s__Lachnospiraceae_bacterium_MA2020	3.2.1.37	GH43_12
g__Clostridium	s__Clostridium_sp._Maddingley_MBC34-26	3.2.1.8	GH11
g__Klebsiella	s__Klebsiella_michiganensis	3.2.1.37	GH43_11
g__Klebsiella	s__unclassified_g__Klebsiella	3.2.1.4	GH8
g__Lachnoclostridium	s__Lachnoclostridium_phytofermentans	3.2.1.4	GH5_4
g__Lactococcus	s__Lactococcus_lactis	3.2.1.37	GH43_11
g__Leclercia	s__Leclercia_adecarboxylata	3.2.1.37	GH43_11
g__Enterobacter	s__Enterobacter_asburiae	3.2.1.4	GH8
g__Bifidobacterium	s__Bifidobacterium_dentium	3.2.1.4	GH5_4
g__Klebsiella	s__Klebsiella_pneumoniae	3.2.1.37	GH43_11
g__Clostridium	s__Clostridium_neonatale	3.2.1.4	GH5_25
g__Paenibacillus	s__Paenibacillus_elgii	3.2.1.4	GH5_2
g__Enterobacter	s__Enterobacter_cloacae	3.2.1.4	GH8
g__Enterobacter	s__Enterobacter_sp._638	3.2.1.4	GH8
g__Klebsiella	s__Klebsiella_variicola	3.2.1.4	GH9
g__Enterobacter	s__Enterobacter_mori	3.2.1.4	GH8
g__Lachnoclostridium	s__[Clostridium]_symbiosum	3.2.1.4	GH5_2
g__Clostridium	s__Clostridium_sp._KLE_1755	3.2.1.37	GH43_11
g__unclassified_f__Enterobacteriaceae	s__unclassified_f__Enterobacteriaceae	3.2.1.4	GH8
g__Clostridium	s__Clostridium_disporicum	3.2.1.8	CBM22
g__Weissella	s__Weissella_sp._92	3.2.1.37	GH43_11
g__Enterobacter	s__unclassified_g__Enterobacter	3.2.1.4	GH8
g__Escherichia	s__unclassified_g__Escherichia	3.2.1.4	GH8
g__Phyllostachys	s__Phyllostachys_edulis	3.2.1.4	GH9
g__Serratia	s__unclassified_g__Serratia	3.2.1.4	GH8
g__Clostridium	s__Clostridium_butyricum	3.2.1.4	GH5_25
g__unclassified_f__Enterobacteriaceae	s__unclassified_f__Enterobacteriaceae	3.2.1.4	GH8
g__Clostridium	s__Clostridium_neonatale	3.2.1.4	GH5_25
g__Clostridium	s__Clostridium_sp._DL-VIII	3.2.1.37	GH39
g__Enterobacter	s__unclassified_g__Enterobacter	3.2.1.4	GH8
g__Lactococcus	s__Lactococcus_lactis	3.2.1.37	GH43_11
g__Raoultella	s__Raoultella_planticola	3.2.1.37	GH43_11

Genus	Species	KEGG_Enzyme	CAZY_Far
g__Klebsiella	s__Klebsiella_oxytoca	3.2.1.4	GH8
g__Clostridium	s__Clostridium_sp._JCC	3.2.1.37	GH43_11
g__Phyllostachys	s__Phyllostachys_edulis	3.2.1.4	GH9
g__Cedecea	s__Cedecea_neteri	3.2.1.4	GH8
g__Raoultella	s__Raoultella_ornithinolytica	3.2.1.37	GH43_11
g__Enterobacter	s__Enterobacter_sp._638	3.2.1.4	GH8
g__Cedecea	s__Cedecea_davisae	3.2.1.4	GH8
g__Clostridium	s__Clostridium_colicanis	3.2.1.37	GH43_11
g__Flavobacterium	s__Flavobacterium_sp._83	3.2.1.37	GH43_12
g__Clostridium	s__Clostridium_butyricum	3.2.1.37	GH43_11
g__Bifidobacterium	s__Bifidobacterium_pseudolongum	3.2.1.37	GH43_11
g__Clostridium	s__Clostridium_disporicum	3.2.1.37	GH43_12
g__Klebsiella	s__Klebsiella_oxytoca	3.2.1.4	GH8
g__Klebsiella	s__Klebsiella_cf._planticola_B43	3.2.1.4	GH8
g__Lactococcus	s__Lactococcus_lactis	3.2.1.37	GH43_11
g__Zea	s__Zea_mays	3.2.1.4	GH9
g__Leclercia	s__Leclercia_adecarboxylata	3.2.1.37	GH43_11
g__Clostridium	s__Clostridium_bornimense	3.2.1.4	CBM13
g__Klebsiella	s__Klebsiella_michiganensis	3.2.1.37	GH43_11
g__Enterobacter	s__Enterobacter_mori	3.2.1.4	GH8
g__Clostridium	s__Clostridium_disporicum	3.2.1.37	GH43_11
g__Brachypodium	s__Brachypodium_distachyon	3.2.1.4	GH9
g__Clostridium	s__Clostridium_neonatale	3.2.1.8	GH10
g__Clostridium	s__Clostridium_colicanis	3.2.1.37	GH43_11
g__Klebsiella	s__Klebsiella_pneumoniae	3.2.1.37	GH43_11
g__Klebsiella	s__unclassified_g__Klebsiella	3.2.1.4	GH8
g__Enterobacter	s__Enterobacter_cancerogenus	3.2.1.4	GH8
g__Serratia	s__Serratia_ureilytica	3.2.1.4	GH8
g__Erysipelatoclostridium	s__[Clostridium]_saccharogumia	3.2.1.37	GH43_11
g__Oryza	s__Oryza_sativa	3.2.1.4	GH9
g__Lactococcus	s__Lactococcus_lactis	3.2.1.37	GH43_11
g__Yersinia	s__Yersinia_massiliensis	3.2.1.4	GH8
g__Klebsiella	s__Klebsiella_pneumoniae	3.2.1.37	GH43_11
g__Raoultella	s__Raoultella_ornithinolytica	3.2.1.37	GH43_11
g__Enterobacter	s__Enterobacter_mori	3.2.1.4	GH8
g__Enterobacter	s__Enterobacter_hormaechei	3.2.1.4	GH8
g__Exophiala	s__Exophiala_spinifera	3.2.1.37	GH3
g__Escherichia	s__Escherichia_sp._TW09276	3.2.1.4	GH8
g__Bifidobacterium	s__Bifidobacterium_dentium	3.2.1.4	GH5_4
g__Streptomyces	s__Streptomyces_purpurogeneiscleroticus	3.2.1.8	GH10
g__Enterobacter	s__Enterobacter_cloacae	3.2.1.37	GH43_11
g__Klebsiella	s__Klebsiella_oxytoca	3.2.1.4	CBM4
g__Enterobacter	s__Enterobacter_cloacae	3.2.1.4	GH8
g__Cedecea	s__Cedecea_neteri	3.2.1.4	GH8
g__Clostridium	s__Clostridium_saccharoperbutylacetonicum	3.2.1.8	GH10
g__Oryza	s__Oryza_sativa	3.2.1.4	GH9
g__Serratia	s__Serratia_fonticola	3.2.1.37	GH43_11
g__Klebsiella	s__Klebsiella_cf._planticola_B43	3.2.1.4	GH8
g__Lactococcus	s__Lactococcus_lactis	3.2.1.37	GH43_11
g__Leclercia	s__Leclercia_adecarboxylata	3.2.1.4	GH8
g__Clostridium	s__Clostridium_cellulovorans	3.2.1.37	GH43_11
g__Setaria	s__Setaria_italica	3.2.1.37	GH3
g__Pluralibacter	s__Pluralibacter_gergoviae	3.2.1.4	GH8
g__Phyllostachys	s__Phyllostachys_edulis	3.2.1.4	GH9
g__Enterobacter	s__Enterobacter_mori	3.2.1.4	GH8

Genus	Species	KEGG_Enzyme	CAZY_Far
g__Shigella	s__Shigella_sonnei	3.2.1.4	GH8
g__Enterobacter	s__Enterobacter_cloacae	3.2.1.4	GH8
g__Clostridium	s__Clostridium_sp._Maddingley_MBC34-26	3.2.1.8	CBM9
g__Sorghum	s__Sorghum_bicolor	3.2.1.4	GH9
g__Kluyvera	s__Kluyvera_cryocrescens	3.2.1.4	GH8
g__Yokenella	s__Yokenella_regensburgei	3.2.1.4	GH8
g__Escherichia	s__Escherichia_coli	3.2.1.4	GH8
g__Oryza	s__Oryza_sativa	3.2.1.4	GH9
g__Oryza	s__Oryza_brachyantha	3.2.1.4	GH9
g__Aegilops	s__Aegilops_tauschii	3.2.1.4	GH9
g__Brachypodium	s__Brachypodium_distachyon	3.2.1.4	GH9
g__Clostridium	s__Clostridium_butyricum	3.2.1.4	GH5_25
g__Clostridium	s__Clostridium_saccharoperbutylacetonicum	3.2.1.37	GH39
g__Enterobacter	s__Enterobacter_cloacae	3.2.1.37	GH43_11
g__Leuconostoc	s__Leuconostoc_lactis	3.2.1.37	GH43_11
g__Clostridium	s__Clostridium_sp._Maddingley_MBC34-26	3.2.1.8	GH10
g__Pediococcus	s__Pediococcus_acidilactici	3.2.1.37	GH43_11
g__Leifsonia	s__Leifsonia_xyli	3.2.1.8	GH10
g__Cedecea	s__Cedecea_davisae	3.2.1.4	GH8
g__Hafnia	s__Hafnia_alvei	3.2.1.4	GH8
g__Serratia	s__Serratia_ureilytica	3.2.1.4	GH8
g__Enterobacter	s__Enterobacter_mori	3.2.1.4	GH8
g__Yokenella	s__Yokenella_regensburgei	3.2.1.4	GH8
g__Klebsiella	s__Klebsiella_cf._planticola_B43	3.2.1.4	GH8
g__unclassified_f__Enterobacteriaceae	s__unclassified_f__Enterobacteriaceae	3.2.1.4	GH8
g__Lactococcus	s__Lactococcus_lactis	3.2.1.37	GH43_11
g__Clostridium	s__Clostridium_butyricum	3.2.1.4	GH5_25
g__Clostridium	s__Clostridium_sp._Maddingley_MBC34-26	3.2.1.8	GH10
g__Weissella	s__unclassified_g__Weissella	3.2.1.37	GH43_11
g__Enterococcus	s__Enterococcus_durans	3.2.1.37	GH43_11
g__Lactobacillus	s__Lactobacillus_hokkaidonensis	3.2.1.37	GH43_11
g__Klebsiella	s__Klebsiella_pneumoniae	3.2.1.37	GH43_11
g__Clostridium	s__Clostridium_disporicum	3.2.1.8	CBM22
g__unclassified_f__Enterobacteriaceae	s__unclassified_f__Enterobacteriaceae	3.2.1.4	GH8
g__Clostridium	s__Clostridium_neonatale	3.2.1.8	CBM9
g__Enterococcus	s__Enterococcus_faecium	3.2.1.37	GH43_11
g__Enterobacter	s__Enterobacter_asburiae	3.2.1.4	GH8
g__Clostridium	s__Clostridium_beijerinckii	3.2.1.8	GH11
g__Enterobacter	s__unclassified_g__Enterobacter	3.2.1.37	GH43_11
g__Leuconostoc	s__Leuconostoc_lactis	3.2.1.37	GH43_11
g__Leclercia	s__Leclercia_adecarboxylata	3.2.1.4	GH8
g__Lactobacillus	s__Lactobacillus_mucosae	3.2.1.37	GH43_11
g__Clostridium	s__Clostridium_butyricum	3.2.1.37	GH43_11
g__Raoultella	s__Raoultella_ornithinolytica	3.2.1.4	GH8
g__Lactococcus	s__Lactococcus_lactis	3.2.1.37	GH43_11
g__Klebsiella	s__Klebsiella_oxytoca	3.2.1.4	GH8
g__Lactococcus	s__Lactococcus_lactis	3.2.1.37	GH43_11
g__Klebsiella	s__Klebsiella_oxytoca	3.2.1.37	GH43_11
g__Cedecea	s__Cedecea_davisae	3.2.1.4	GH8
g__unclassified_f__Enterobacteriaceae	s__unclassified_f__Enterobacteriaceae	3.2.1.4	GH8
g__Lactococcus	s__Lactococcus_lactis	3.2.1.8	GH11
g__Oryza	s__Oryza_brachyantha	3.2.1.4	GH9
g__Aegilops	s__Aegilops_tauschii	3.2.1.4	GH9

Supplementary Table S8: The relative value of contribution to polysaccharide degradation within the high carbohydrate-metabolizing and dominant species.

Function	Taxon	YS1_S60	BS1_S62	YS2_S61	BS2_S58	YS3_S57	BS3_S59
3.2.1.4	others	0.7902	0.45987	1.8715	0.76702	2.02833	2.04369
3.2.1.4	s__unclassified_f__Enterobacteriaceae	3.20434	2.95893	4.91857	3.68856	0.8989	2.61309
3.2.1.4	s__Escherichia_coli	1.03906	0.82813	1.48438	0.96875	0.20313	0.72656
3.2.1.4	s__Shigella_sonnei	0.04826	0.00536	0.03217	0.02681	0	0.04826
3.2.1.4	s__Raoultella_ornithinolytica	0	0	0.10101	0.22511	0.00577	0.00866
3.2.1.4	s__Clostridium_butyricum	0.008	0.42237	0	0	0	0
3.2.1.4	s__Klebsiella_pneumoniae	0.00733	0.01099	0.00366	0.00733	0	0
3.2.1.4	s__Enterobacter_cloacae	0.0119	0	0.10601	0.02754	0	0
3.2.1.8	others	0	0	0.13384	0.00355	0.01445	0.14192
3.2.1.8	s__Clostridium_disporicum	0.00223	0.07466	0.00187	0	0.00312	0.07518
3.2.1.8	s__Lactococcus_lactis	0	0	0.00398	0.01193	0.01193	0.02386
3.2.1.37	s__Leuconostoc_lactis	0	0	2.10191	0.20518	0.2246	0.99247
3.2.1.37	s__Clostridium_sp._JCC	0.01091	0.15515	0.02788	0.00727	0.15515	0.33212
3.2.1.37	s__Lactococcus_lactis	0.03072	0.03205	0.39725	0.17758	0.03926	0.3129
3.2.1.37	others	0.42695	2.22074	0.35383	0.26759	0.01744	0.30723
3.2.1.37	s__Clostridium_disporicum	0.00768	0.36298	0.00319	0.00837	0	0.28619
3.2.1.37	s__Streptococcus_pneumoniae	0	0	0.04137	0.02246	0.00118	0.0721
3.2.1.37	s__Clostridium_colicanis	0	0.07141	0.00358	0	0	0.02993
3.2.1.37	s__Raoultella_ornithinolytica	0.00663	0.00187	0.30849	0.36244	0.00375	0.00681
3.2.1.37	s__Clostridium_butyricum	0.00772	0.08475	0.01136	0	0	0.00772
3.2.1.37	s__Klebsiella_pneumoniae	0.06297	0.0641	0.01046	0.00393	0	0.0074
3.2.1.37	s__Streptococcus_pasteurianus	1.56117	0.13204	0.03107	0.05437	0	0.00129
3.2.1.37	s__Lactobacillus_mucosae	0.2619	1.55833	0.00238	0	0	0
3.2.1.37	s__Enterococcus_faecium	0.04461	0.23709	0.00892	0.05226	0	0
3.2.1.37	s__Enterobacter_cloacae	0.23894	0.02212	0.03852	0.03982	0	0
3.2.1.37	s__Pediococcus_acidilactici	0.02433	0	0	0	0	0

Supplementary References

Lu HY, Song M, Shi WD, Guo T. Anthrone Photometric on the determination of sodium carboxymethyl cellulose. *J Tianjin Univ Light Ind* (1996) **2**: 86-88. (In Chinese)

Fan PC, Tian J, Huang JM, Lei WQ, Qiu HD. On the determination of cellulose and lignin of peanut shells. *J Chongqing Univ Sci Technol:Nat Sci Ed* (2008) **10**: 64-65. (In Chinese)

Horn SJ, Vaaje-Kolstad G, Westereng B, Eijsink VG. Novel enzymes for the degradation of cellulose. *Biotechnology for Biofuels* (2012) **5**: 45.

Grady EN, Macdonald J, Liu L, Richman A, Yuan ZC. Current knowledge and perspectives of *Paenibacillus*: A review. *Microbial Cell Factories* (2016) **15**:203.

Wu F, et al. The study on rearing and health monitoring technology of giant pandas in low altitude area. Shanghai: Shanghai Wild Animal Park Development Co, Ltd, (2018).

Supplementary Figure 1

(A)

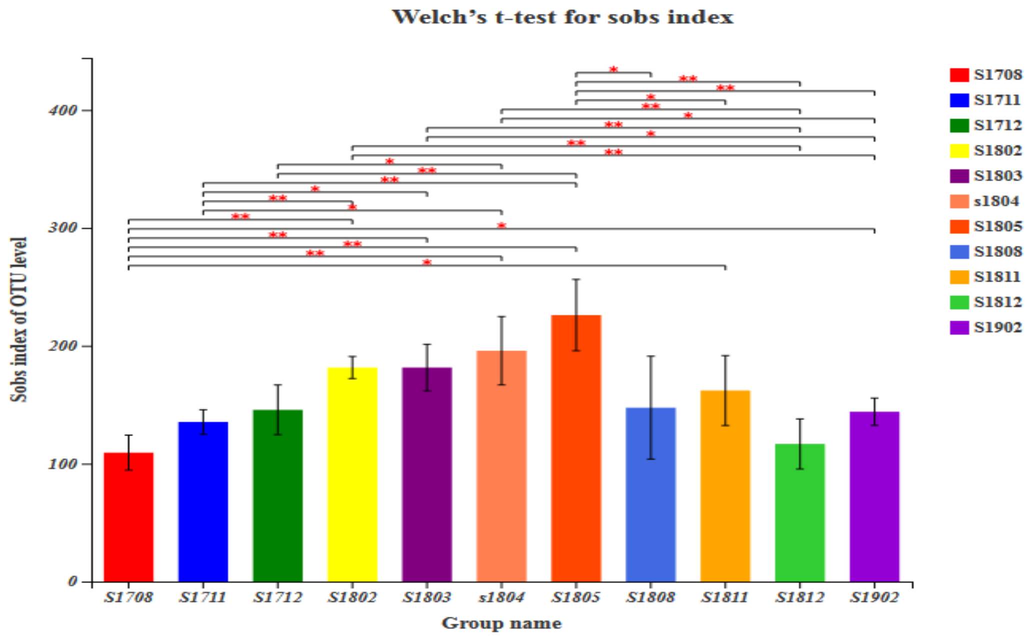

(B)

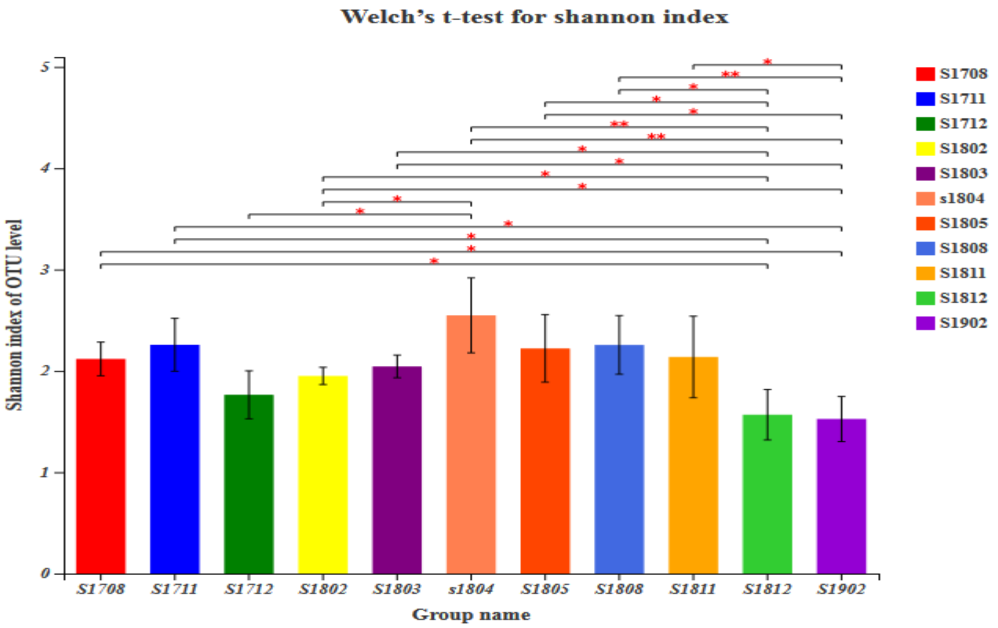

Supplementary Figure 1 Significant changes of bacterial richness and diversity in the growth of giant panda. Different colorful bars represent twin panda fecal samples' bacterial diversity indices at different sampling time and were expressed by mean ± standard error. Abscissa was S (Sample) + year (last two digits) + month (two digits). (A) Significant differences of the total number of species observed (Sobs) between either two groups; (B) Significant differences of Shannon's index between either two groups of bacteria. Welch's t-test, * P<0.05, ** P<0.01, *** P<0.001

Supplementary Figure 2

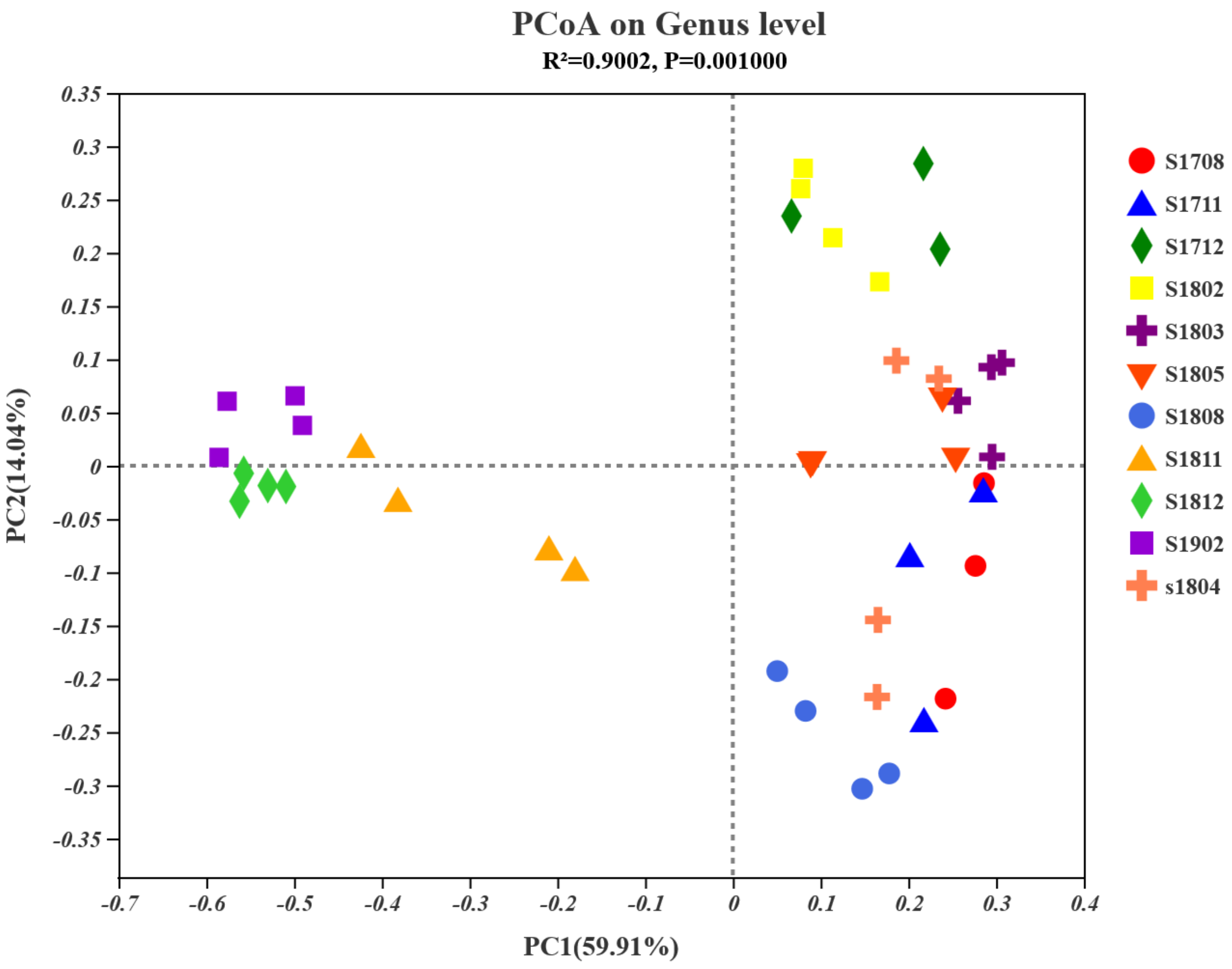

Supplementary Figure 2 Bacterial Principal coordinates analysis (PCoA) on genus level of twin panda cubs during dietary shift stage. PCoA analysis was based on Bray-Curtis values. X-axis and Y-axis represent the two selected main axes which percentage indicated the value explains the difference of sample composition in each main axis; the scales of the X-axis and Y-axis are relative distances and have no practical significance; points of different colors and shapes represent samples which were sorted by sampling period and named as S (Sample) + year (last two digits) + month (two digits). The closer the two sample points, the more similar the species composition of the two samples.

Supplementary Figure 3

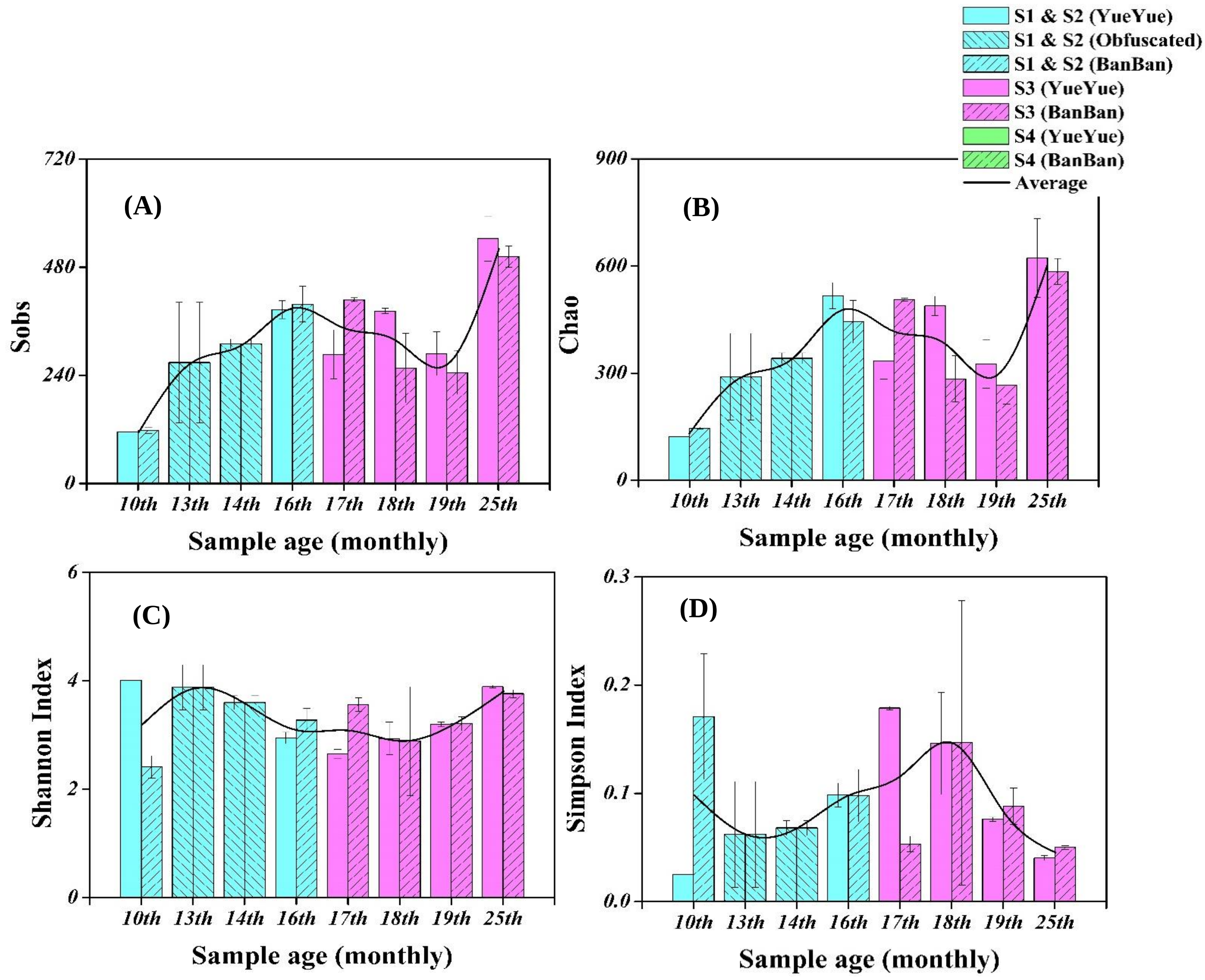

Supplementary Figure 3 Fecal fungal alpha-diversity in giant panda cubs. The colorful bars represent the different stages during dietary shifts. The blue bars represent individual diversity indices in Stage 1 and Stage 2 (formula and biting bamboo); the purple bars represent those in Stage 3 (bamboo consumption) and Stage 4 (bamboo diet). Results were expressed by mean ± standard error. Black trend lines represent the average of the two study individuals. Intestinal fungal alpha diversity is reflected by height of column of each sampling time and the overall fluctuations in four indices are expressed by the black trend line. (A) Sobs; (B) Chaos; (C) Shannon index; (D) Simpson index about intestinal fungi. Due that the two cubs live in together, increasing difficulty in fecal collection, the feces at 13th and 14th couldn’t be distinguished and treated as mixed samples.

Supplementary Figure 4

(A)

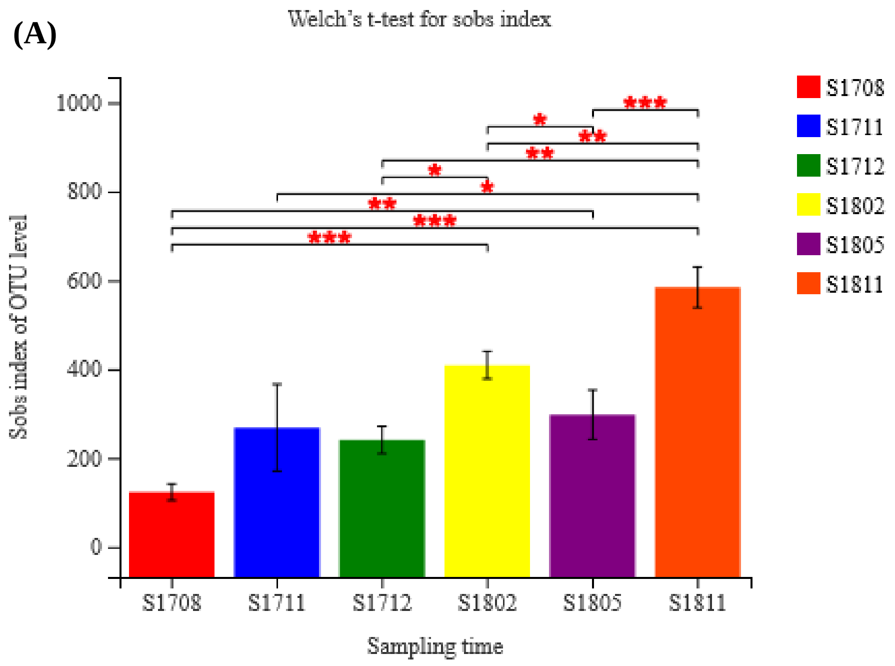

(B)

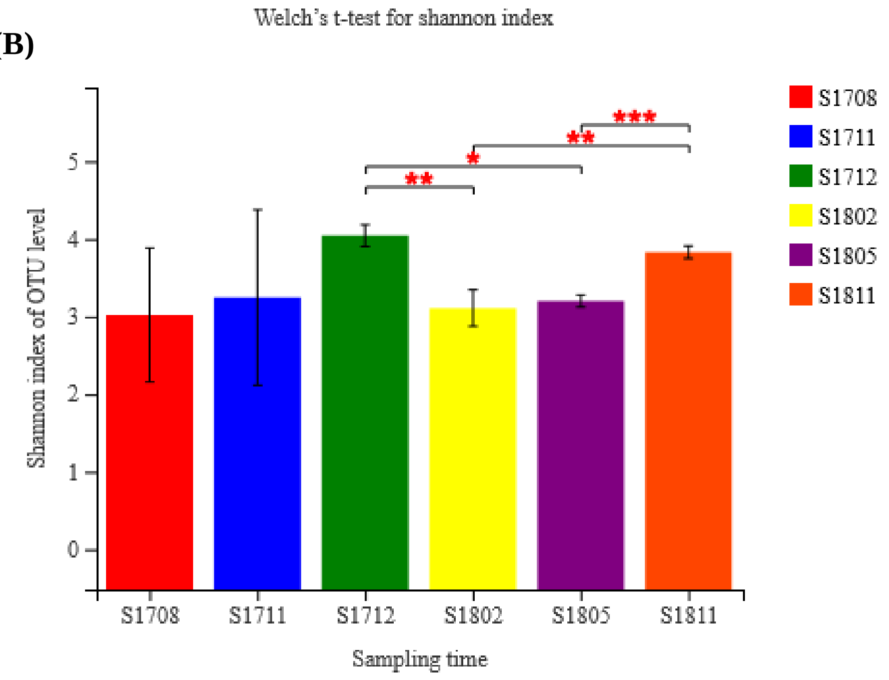

Supplementary Figure 4 Significant changes of fugal abundance and diversity in the growth of giant panda. Different colorful bars represent twin panda fecal samples' fungal diversity indices at different sampling time and were expressed by mean ± standard error. Abscissa was S (Sample) + year (last two digits) + month (two digits). (A) Significant differences of Sobs between either two groups; (B) Significant differences of Shannon's index between either two groups of fungi. Welch's t-test, * P<0.05, ** P<0.01, *** P<0.001.

Supplementary Figure 5

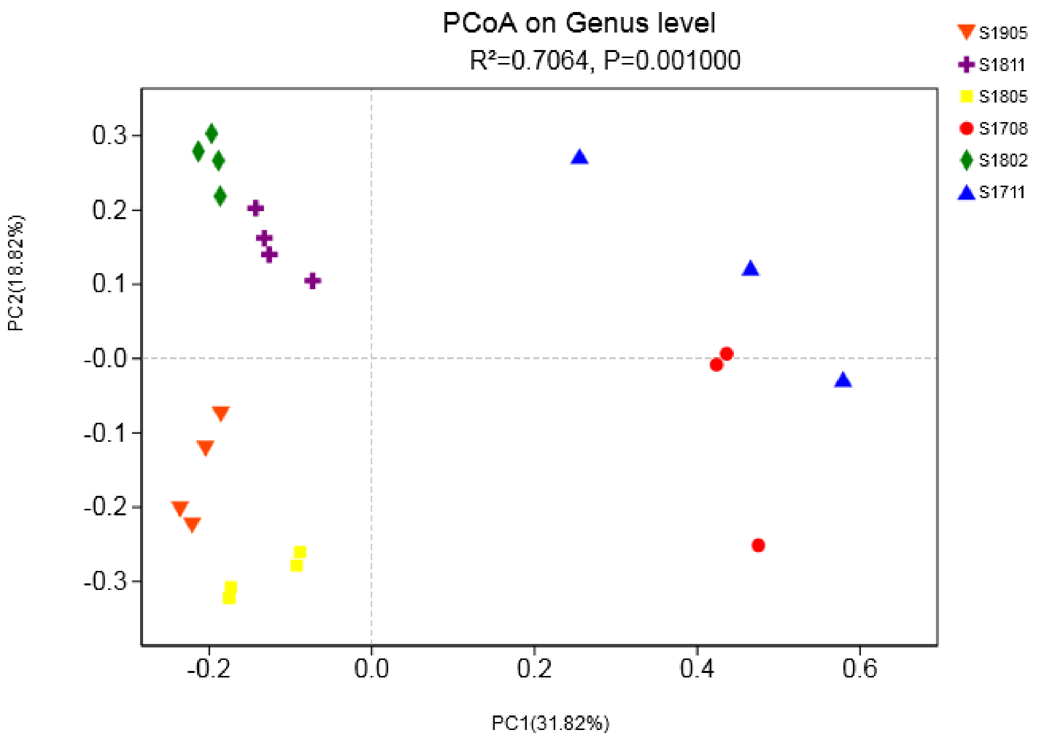

Supplementary Figure 5 Fugal PCoA analysis on genus level of twin panda cubs during dietary change stage. PCoA analysis was based on Bray-Curtis values. X-axis and Y-axis represent the two selected main axes which percentage indicated the value explains the difference of sample composition in each main axis; the scales of the X-axis and Y-axis are relative distances and have no practical significance; points of different colors and shapes represent samples which were sorted by sampling period and named as S (Sample) + year (last two digits) + month (two digits). The closer the two sample points, the more similar the species composition of the two samples.

Supplementary Figure 6

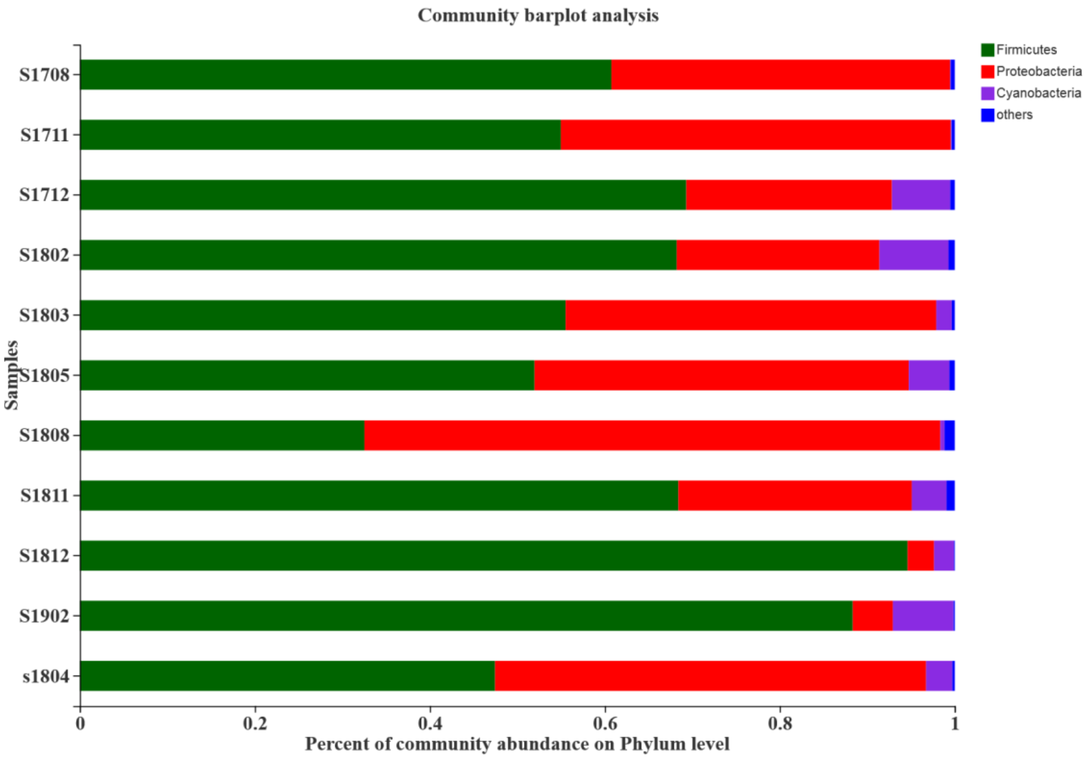

Supplementary Figure 6 Bar-plot analysis of bacterial compositions at phylum in the growth of panda cubs. The community bar-plot analysis was based on the average of two individuals and represents the overall conditions of bacterial composition in every sampling time. Different colors represent different taxa at phylum level and length are proportional to the abundance of each taxa.

Supplementary Figure 7

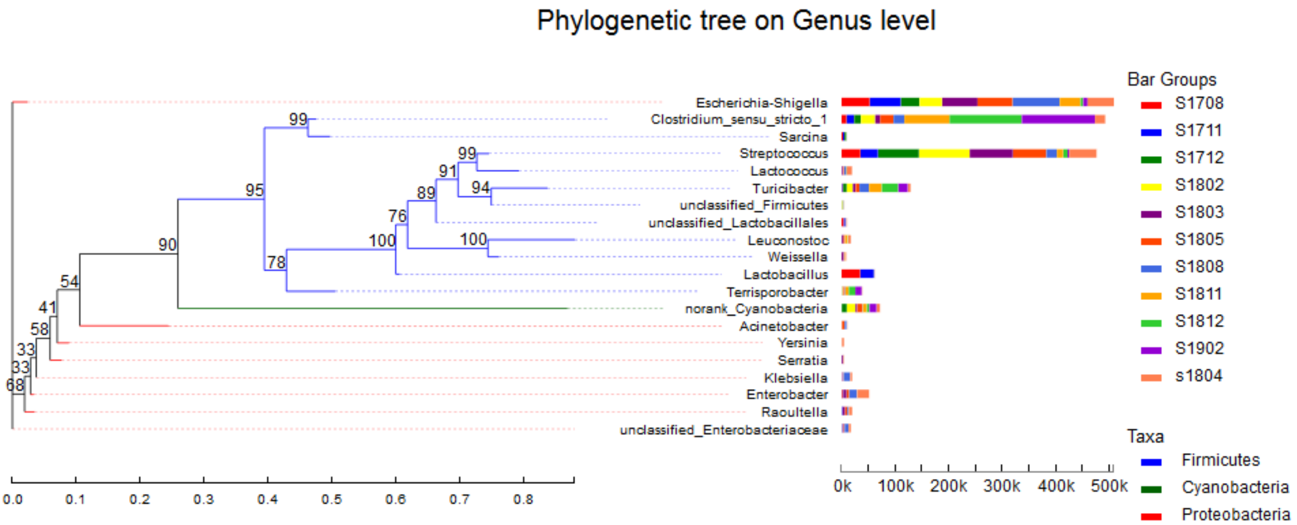

Supplementary Figure 7 Component of fecal bacterial structure in giant panda cubs. Phylogenetic tree at genus level of bacteria based on the 20 most abundant taxa in YueYue and BanBan. Each branch in the tree represents a type of genus. The branches were colored according to the taxonomic level of the phylum. The length of the branch is the evolutionary distance between two genera. The histogram on the right show the proportion of taxon reads in different sampling times.

Supplementary Figure 8

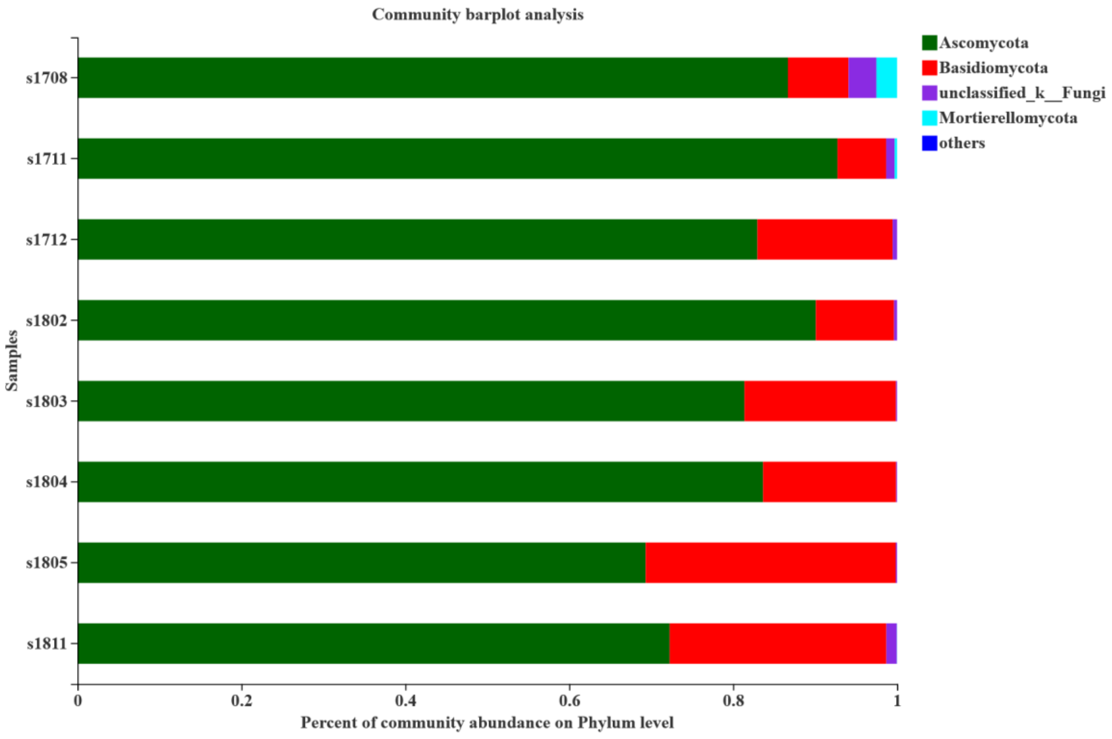

Supplementary Figure 8 Bar-plot analysis of fungal compositions at phylum in the growth of panda cubs. The community bar-plot analysis was based on the average of two individuals and represents the overall conditions of fungal composition in every sampling time. Different colors represent different taxa at phylum level and length are proportional to the abundance of each taxa.

Supplementary Figure 9

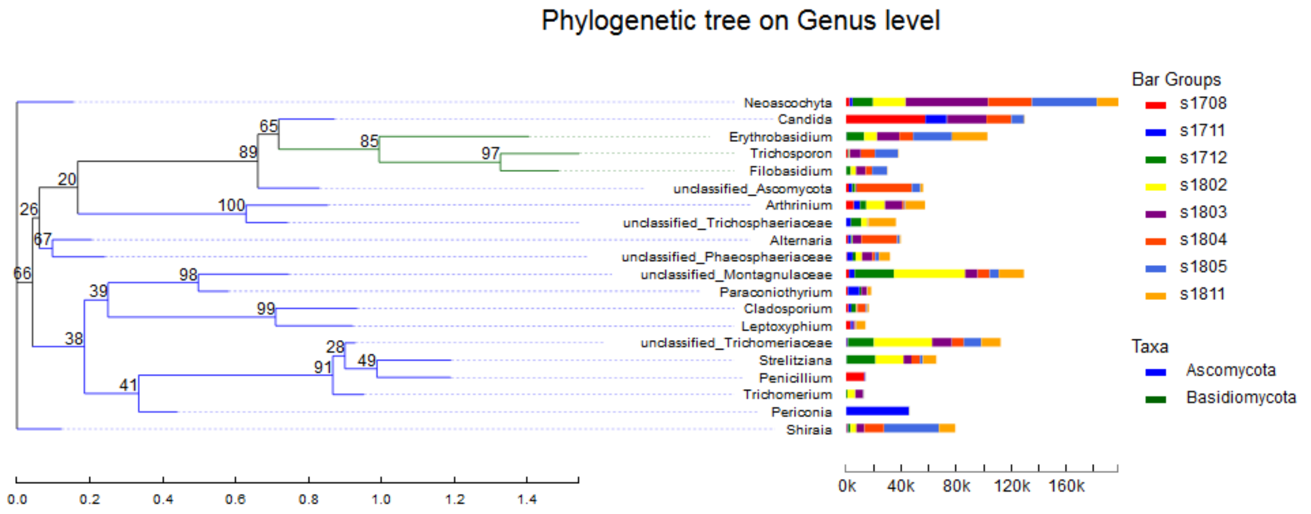

Supplementary Figure 9 Component of fecal fungal structure in giant panda cubs. Phylogenetic tree on genus level of fungi based on 20 most abundant taxa in YueYue and BanBan. Each branch in the tree represents a type of genus. The branches were colored according to the taxonomic level of the phylum. The length of the branch is the evolutionary distance between two genera. The histogram on the right shows the proportion of taxon reads in different sampling times.

Supplementary Figure 10

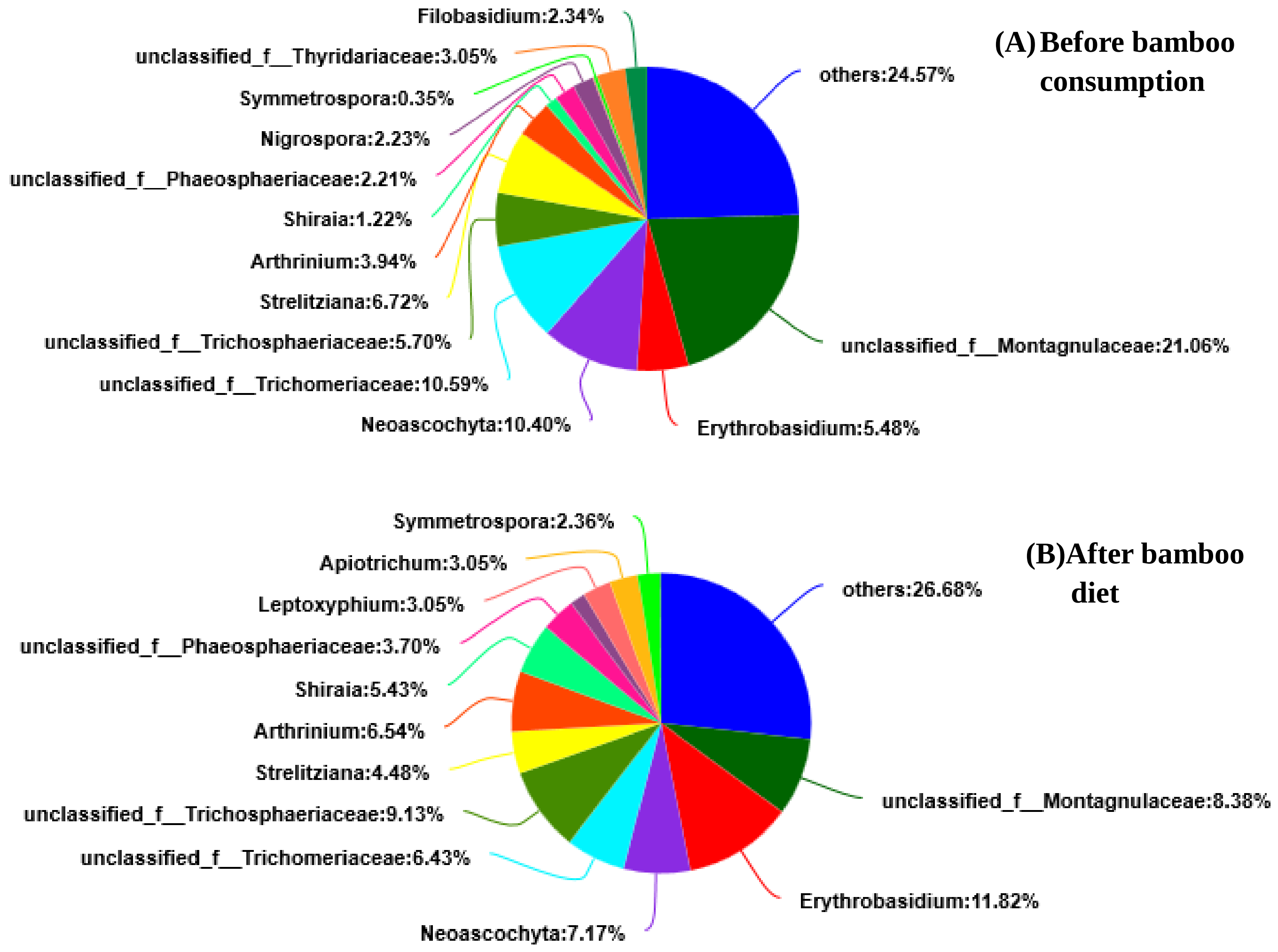

Supplementary Figure 10 Fungal community before and after bamboo consumption in pie chart. Pie charts show the percentage of the dominant taxa in fungal community. Each color represents a type of fungi taxa at genus level. The larger the color block area, the more the richness. Blocks in the same color represent the same genus, the relative abundance of each taxa can be compared by the block area, not absolute value.

Supplementary Figure 11

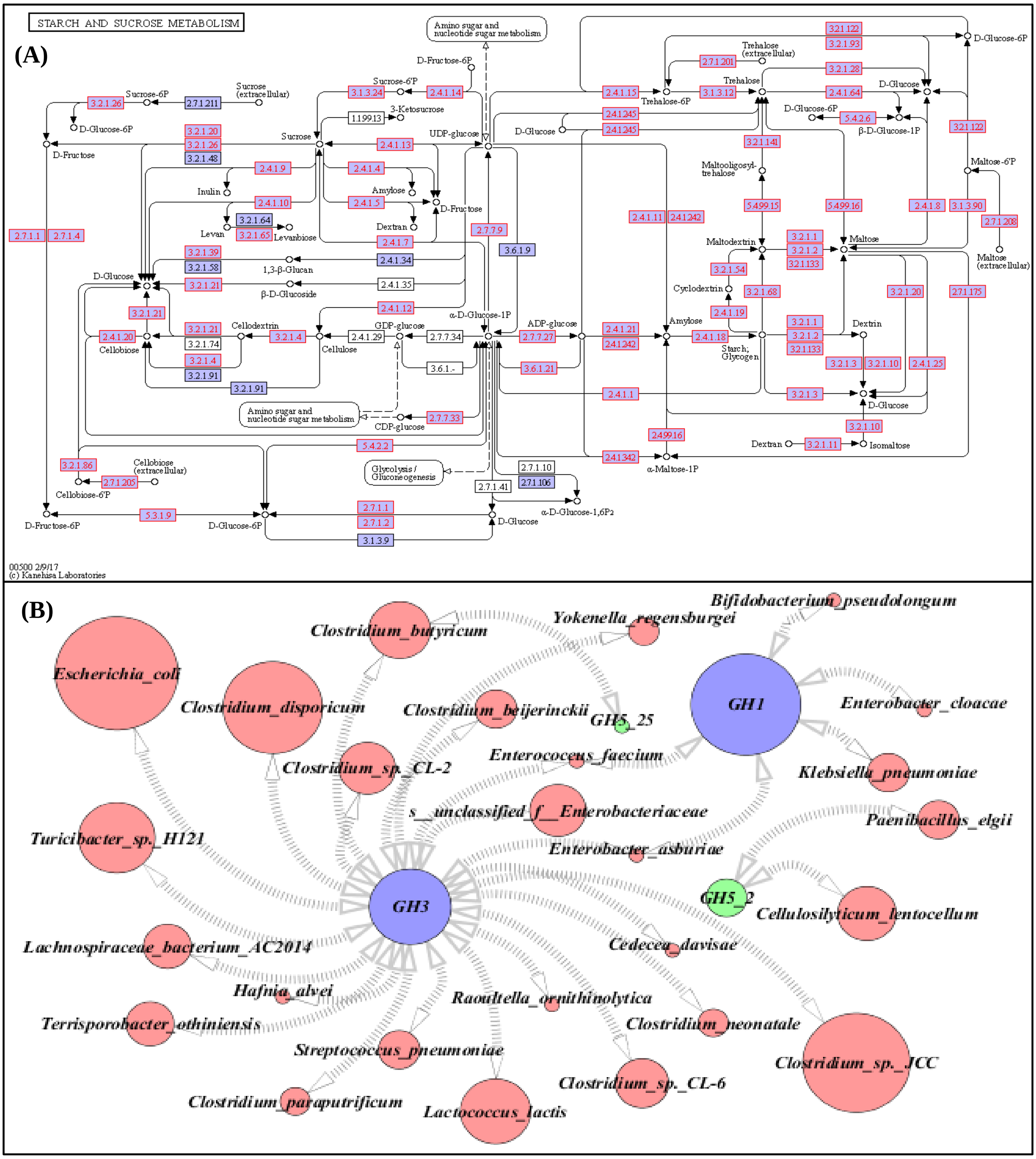

Supplementary Figure 11 The network analysis between specific cellulose degradation pathway and related dominant bacteria in giant pandas with bamboo diet. (A) Cellulose degradation pathway contained in ko00500 pathway. The key enzymic node whose outline was painted in red represents that genes encoding it can be found in gut microbial genome of giant panda, otherwise whose outline in black are

with rare or none corresponding genes, and one metabolic pathway may be active in microbiome with the enzyme nodes on it all being red. (B) The network analysis between cellulose degradation and related dominant bacteria (> 25 species) in giant pandas with bamboo diet. The potential activity of two key enzymes including Endoglucanase (EC3.2.1.4) corresponding to GH5_2 and GH5_25, and β-glucosidase (EC 3.2.1.21) corresponding to GH1 and GH3 genes were calculated with related genes abundance and filled in green and blue, relatively. The more the genes, the bigger the circle. Those circles representing dominant bacteria were filled in red, and the more abundance the species, the bigger the circle. The correlation between functions and bacteria species was linked with grey arrows.

Supplementary Figure 12

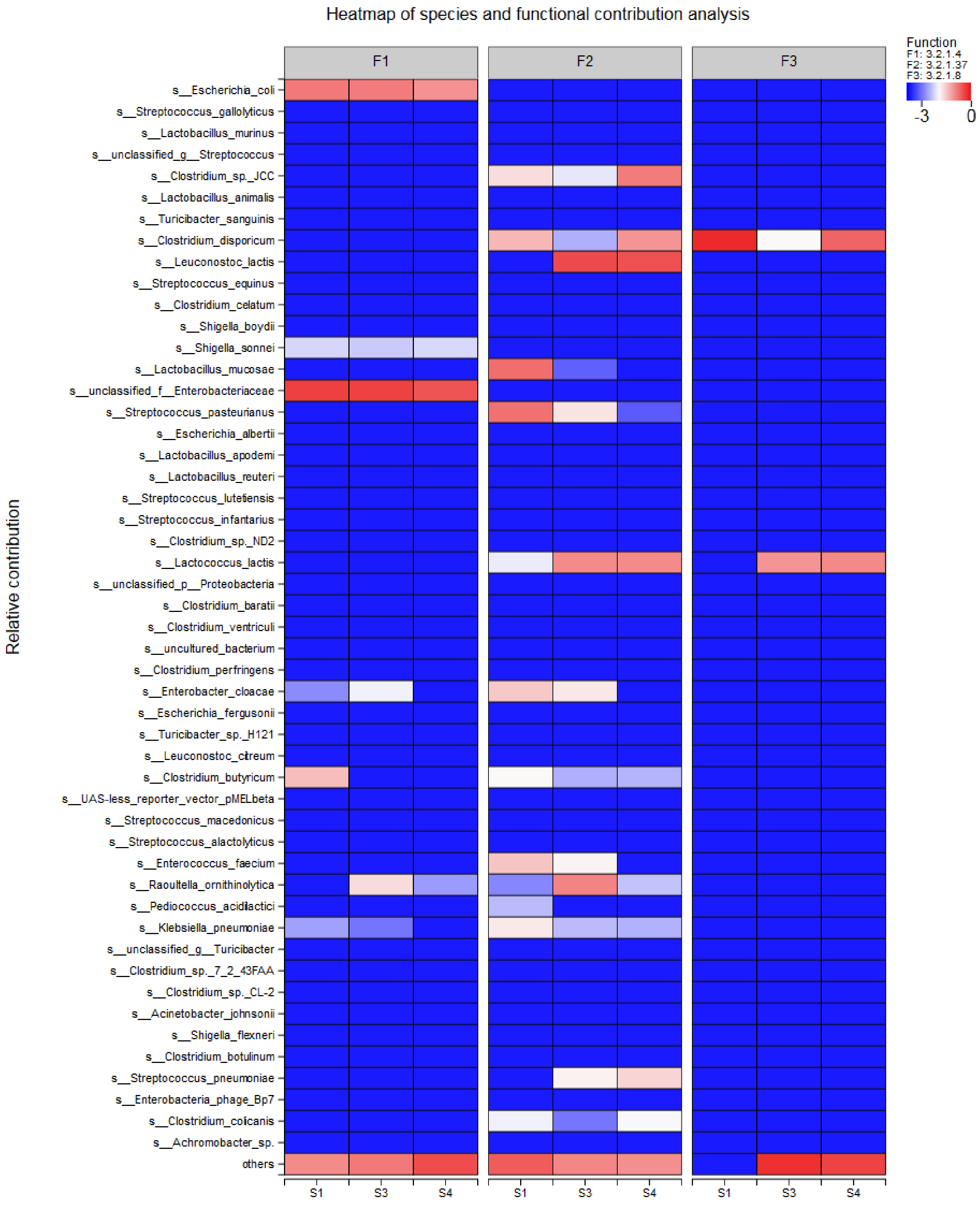

Supplementary Figure 12 Contribution of dominant bacteria on cellulose and hemicellulose metabolism. The dominant species that actively participate in carbohydrate-metabolism on the top of

50 were list in heat map. Three pieces from left to right relatively belong to EC 3.2.1.4, EC3.2.1.37 and EC 3.2.1.8. The abscissa represents different dietary stages (S1, S3 and S4) corresponding to the contribution of species to specific function in specific stage. The deeper the red, the higher the contribution of species. The deeper the blue, the lower the contribution of species, even no contribution.
